# Deciphering reductive dehalogenase specificity through targeted mutagenesis of chloroalkane reductases

**DOI:** 10.1128/aem.01501-24

**Published:** 2025-02-13

**Authors:** Katherine J. Picott, Connor M. Bowers, Elizabeth A. Edwards

**Affiliations:** 1 Department of Chemical Engineering and Applied Chemistry, University of Toronto 686119 , Toronto, Ontario, Canada; 2 Department of Cell and Systems Biology, University of Toronto 98684 , Toronto, Ontario, Canada; Universidad de los Andes, Bogotá, Colombia

**Keywords:** bioremediation, reductive dehalogenase, mutagenesis, *Dehalobacter*, chloroalkane, substrate specificity, active site

## Abstract

Reductive dehalogenases (RDases) are essential in the anaerobic degradation of various organohalide contaminants. This family of enzymes has broad sequence diversity, but high structural conservation. There have been few studies assessing how RDase amino acid sequences affect their substrate selectivity. Here, we focus on two chloroalkane RDases, CfrA and DcrA, which have 95% protein sequence identity but have diverged to have opposite substrate preferences. CfrA dechlorinates chloroform (CF) and 1,1,1-trichloroethane (TCA) but not 1,1-dichloroethane (DCA), while DcrA will dechlorinate 1,1-DCA but not CF or 1,1,1-TCA. We mutated several residues in the active site of CfrA to investigate a change in substrate preference and to identify which wild-type residues contribute the most to substrate specialization. We determined that no individual residue solely dictates substrate discrimination, but both Y80W and F125W mutations were needed to force CfrA to prefer 1,1-DCA as a substrate. When using 1,1,2-TCA as a substrate, CfrA predominately performs hydrogenolysis to 1,2-DCA, yet the introduction of the double mutant changed this preference to dihaloelimination (forming vinyl chloride). We use predictive protein models and substrate docking to predict what interactions are made between the enzyme and substrate to aid in selection. The residues of significance identified in this study are consistent with those identified from chloroethene RDases, suggesting residue locations with a particularly high impact on activity.

**IMPORTANCE:**

Reductive dehalogenases (RDases) play an integral role in the removal of chlorinated solvents from the environment. These enzymes have specificity toward different chlorinated compounds, and it is known that natural variants of highly similar RDases can have distinct activities. How specific differences in protein sequence influence activity is largely unknown. In this study, we demonstrate that mutating a few residues within the active site of CfrA—a chloroform and trichloroethane-specific dehalogenase—changes its substrate preference to dichloroethane. We determine that only two mutations are needed to disrupt the native activity, underscoring the nuances in substrate-structure relationships in RDases. Though we are still far from predicting function from the sequence, this knowledge can give some insight into engineering RDases for new target contaminants.

## INTRODUCTION

Organohalide-respiring bacteria (OHRB) are well-known for their role in the bioremediation of chlorinated solvents such as chloroethenes and chloroethanes, many of which are on the priority substance list for toxic compounds (1). OHRB utilize organohalides as their terminal electron acceptor, which are reduced by membrane-bound reductive dehalogenases (RDases) in the electron transport chain (2). RDases catalyze the cleavage of halogen-carbon bonds during the reduction reaction (2). This cleavage can occur via hydrogenolysis, where the halogen is replaced with a proton, or through dihaloelimination (a β-elimination reaction) (2). RDases require a cobamide and two [4Fe-4S] clusters as cofactors for catalysis (3, 4), all of which participate in the reduction of the substrate.

RDases are not unique to OHRB, but are a vast family of enzymes that are found in many different bacteria and even archaea (5, 6). Accordingly, there is high variation in protein sequences; for example, characterized perchloroethene (PCE) dechlorinating RDases from different organisms can have less than 20% pairwise amino acid sequence identity (PID) with each other but maintain the same function (7–13). This high variation makes predicting RDase activity from sequence alone very difficult. An organizational system, the ortholog group (OG) classification, was developed to help keep track of functionally characterized RDases and relate new sequences to potential substrates based on sequence similarity (14, 15). RDases are grouped into OGs based on having >90% PID between all members of the group (14, 15). While these OGs do indeed group RDases that have functions on similar classes of compounds, the exact substrate specificity of RDases is even more discriminating.

OG 97 contains seven characterized chloroalkane reductases (all have >93% PID) that have shown varying substrate preferences among a common group of chlorinated ethanes and methanes (16–21). These enzymes are CfrA (*Dehalobacter* sp. CF), DcrA (*Dehalobacter* sp. DCA), CtrA (*Desulfitobacterium* sp. PR), TmrA (*Dehalobacter* sp. UNSWDHB), ThmA (*Dehalobacter* sp. THM1), RdhA D8M_v2_40029 (*Dehalobacter* sp. 8M), and AcdA (*Dehalobacter* sp. SAD) (16, 18–23). The starkest difference in selectivity comes from CfrA and DcrA, two enzymes that were identified from two closely related *Dehalobacter* in the ACT-3 mixed culture enriched on 1,1,1-trichloroethane (1,1,1-TCA) (16, 24, 25). Rather than competing for the same substrates, CfrA became specialized in 1,1,1-TCA and chloroform (CF) dechlorination, while DcrA primarily dechlorinates CfrA’s product 1,1-dichloroethane (1,1-DCA) (16). Further, when given 1,1,2-trichloroethane (1,1,2-TCA) as a substrate CfrA and DcrA will undergo two different transformation mechanisms (26). CfrA predominantly performs hydrogenolysis to 1,2-dichloroethane (1,2-DCA) and DcrA prefers dihaloelimination to vinyl chloride (VC) (26). Similar cooperative enzyme functions were also observed from unenriched field samples at a 1,1,1-TCA contaminated site (27). The natural evolution of these enzymes with high sequence identity yet contrasting functions is an excellent opportunity to identify key residues that influence substrate preferences and gives a glimpse into the target areas for positive selection.

There have only been three RDase crystal structures solved to date: PceA from *Sulfurospirillum multivorans* (referred to as PceA_S_ from here on) (3)*,* the cytosolic NpRdhA from *Nitrareductor pacificus* pht-3B (4), and most recently PceA (referred to as PceA_D1_) from *Desulfitobacterium hafniense* TCE1 (PDB IDs: 4UQU, 4RAS, and 8Q4H, respectively) (28). These enzymes are all distinct from each other (<30% PID), and share no substrates in common with OG 97. The central fold of RDases, made up of α-helices and β-sheets, is highly conserved and researchers have been able to glean information on potentially important and influential residues; the residues we discuss here are numbered with respect to PceA_S_. Two active site amino acids—Tyr246 and Arg305—were identified in the PceA_S_ crystal structure and were subsequently demonstrated to be essential catalytic residues through mutagenesis (3, 4, 29). These two residues are highly conserved among RDases and are predicted to be involved in the final protonation step of dehalogenation aided by residue Asn272 (3, 4, 29). Additional residues have been proposed to be involved in substrate selection based on either *in silico* analysis or through mutagenesis studies (29–31). One mutagenesis study in *Desulfitobacterium* found several residues—corresponding to Trp96, Thr242, and Trp376 in PceA_S_—in the active sites of both PceA from *D. hafniense* Y51 (referred to as PceA_D2_ going forward) and DcaA from *D. dichloroeliminans* DCA1 (88% PID between PceA_D2_ and DcaA) that modify the size of the substrate preferred by the enzymes when mutated (31). Additionally, Phe38 lines the active site and is proposed to help orient the substrate for catalysis, though this has never been tested (29, 30). These residues are all intriguing targets for assessing substrate preference divergence between CfrA and DcrA. Table 1 compares these corresponding residues along with descriptions of potential functions based on literature.

**TABLE 1:** Residues of interest identified in previous studies on PceA_S_ and DcaA and their putative roles

PceA_S_ (*S. multivorans*)	DcaA (*D. dichloroeliminans* DCA1)^*a*^	CfrA (*Dehalobacter* sp. CF)	DcrA (*Dehalobacter* sp. DCA)	Proposed role/DcaA mutagenesis observations	References
F38	F31	Y80	W80	Lines substrate channel and active site to orient substrate, no mutagenesis performed	(29, 30)
W96	W118	F125	W125	Active site formation, W118F lost activity	(29, 31)
T242	T294^*b*^	Y256	Y256	Substrate interaction, T294V shifted activity toward smaller substrates	(28, 30, 31)
Y246	Y298	C260	Y260	Catalytic residue and suggested proton donor or stabilizing residue, Y298F eliminated hydrogenolysis activity but enhanced dihaloelimination	(3, 4, 29–33)
N272	N324	S286	A286	Stabilizes catalytic residues, no mutagenesis performed	(3, 29–33)
R305	R357	R319	R319	Catalytic residue suggested as a proton donor or stabilizing residue, R357X decreased activity on chloroethenes but increased on tribromoethene and dihaloelimination	(3, 29–33)
W376	W432	M391	W391	Lines substrate channel and active site to orient substrate, W432F decreased activity on chloroethenes but increased on tribromoethene and dihaloelimination	(29, 31)

^*a*^ Residues are consistent in PceA_D2_ from *D. hafniense* Y51.

^*b*^ V294 in PceA_D2_.

In this study, our aim is to broaden our knowledge of RDase-substrate interactions beyond haloethenes. By investigating natural active site variation between the chloroalkane-reductases CfrA and DcrA, we seek to expand our understanding of how RDases fine-tune their specificity. Through site-directed mutagenesis of select residues, we endeavor to identify trends in how amino acid characteristics influence substrate selectivity and preferred transformation pathways. This analysis was enabled by recent developments in heterologous expression of RDases in *Escherichia coli* by co-expression with the vitamin B_12_ uptake pathway proteins (26). We demonstrated that two key mutations were required to effectively transplant the active site from DcrA to CfrA, reversing substrate preferences.

## RESULTS AND DISCUSSION

### Active site transplant rationale

**Fig 1:**
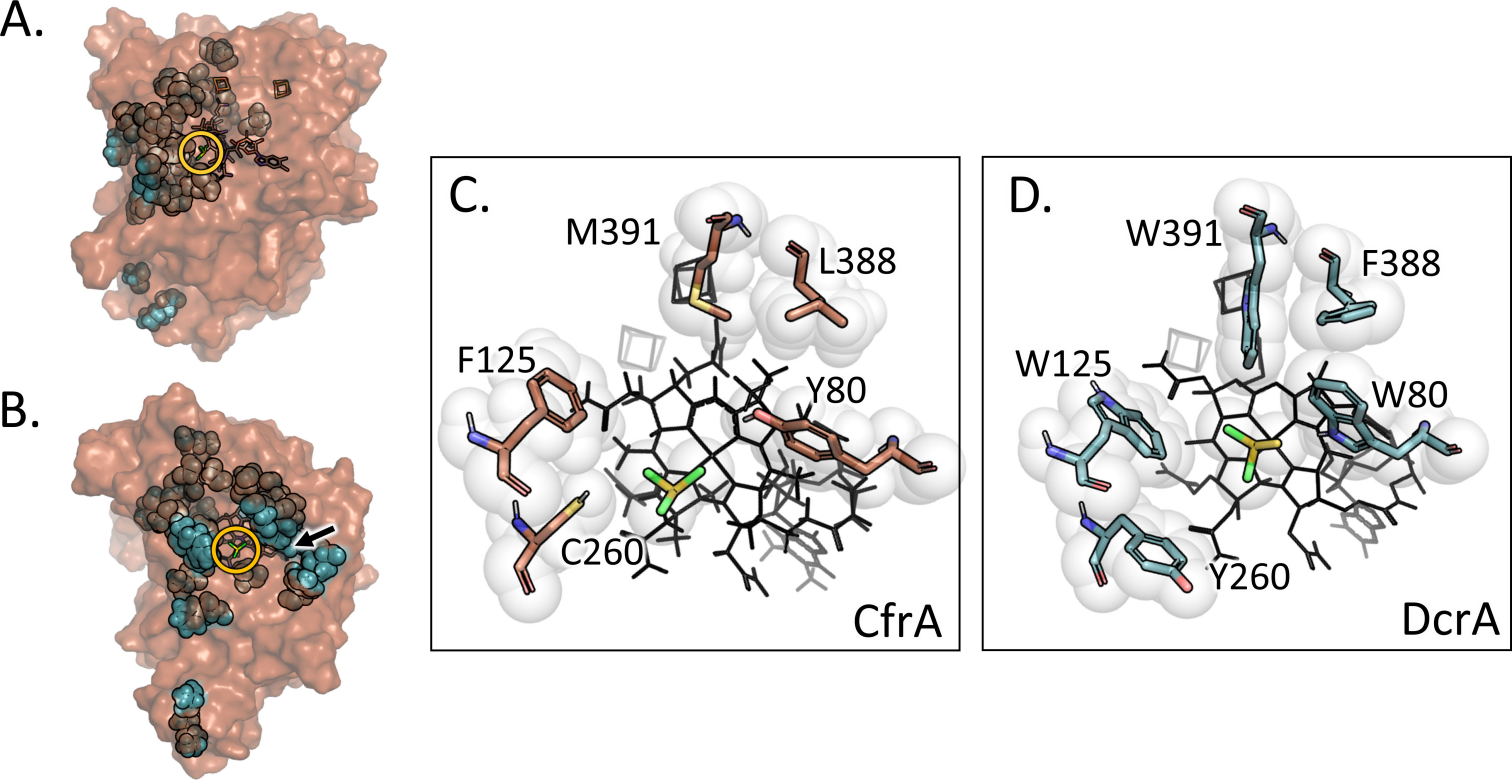
Surface model of CfrA in transparent orange with the amino acids that differ from DcrA shown as spheres and chloroform docked in the active site. (**A**) Profile and (**B**) face view of the active site. The yellow circle indicates the location of the substrate. The black arrow indicates the substrate access channel. The substrate binding face showing the five targeted active site residues in (**C**) CfrA (orange) docked with chloroform and (**D**) DcrA (blue) docked with 1,1-dichloroethane. Substrates are shown with yellow backbones and cobalamin and [4Fe-4S] cofactors are shown as black wire structures.

Previous studies probing the roles of specific residues have focused on PceA_S_ and enzymes that target haloethenes like PCE (29–32), yet many of the key residues established in these studies are variable within OG 97 and specifically between CfrA and DcrA. When structurally modeled, it is apparent that the differences between CfrA and DcrA are localized in the active site, particularly in the substrate access channel and binding pocket (Fig. 1A and B). Phillips et al. suggested five residues within the putative active site that may influence the activity and the mechanism of these chloroalkane reductases (34). Three of these residues at positions 80, 260, and 391, align structurally with Phe38, Tyr246, and Trp376 of PceA_S_, respectively. Residues 262 and 388 were also proposed to have functional significance in OG 97, but residue 262 is identical in both CfrA and DcrA so it will not be explored here (34). Additionally, the mutation of Trp118 in DcaA from *D. dichloroeliminans* has been shown to have a detrimental effect on its activity, and the equivalent residues 125 in CfrA and DcrA diverge (31). Therefore, we have chosen five variable active site residues to mutate in CfrA and DcrA based on previous analyses and proposed roles: residues 80, 125, 260, 388, and 391. All of these residues differ between CfrA and DcrA, but the rest of OG 97 members have varying combinations of these residues, which might contribute to their more flexible substrate selection (Table 2). A full alignment of characterized OG 97 RDases is in Supplemental Text S1.

**TABLE 2:** Active site residues in all characterized OG 97 members and their relative activity (based on culture activity or enzyme assays) on known substrates^*c*^

	Active site residue					Relative activity level			
Enzyme	80	125	260	388	391	High (full reduction or >50% of top enzyme rate)	Medium (incomplete reduction or 5–50% of top enzyme rate)	Low to none (trace or no reduction or <5% of top enzyme rate)	References
Consensus (organism)	F	F	X	F	W				
CfrA (*Dehalobacter* sp. CF)	Y	F	C	L	M	CF; 1,1,1-TCA; 1,1,2-TCA→ 1,2-DCA		1,1-DCA; 1,1,2-TCA→ VC	(16, 26)
DcrA (*Dehalobacter* sp. DCA)	W	W	Y	F	W	1,1-DCA; 1,1,2-TCA→ VC		CF; 1,1,1-TCA; 1,1,2-TCA→ 1,2-DCA	(16, 26)
TmrA (*Dehalobacter* sp. UNSWDHB)	F	F	F	F	W	CF; 1,1,2-TCA→ 1,2-DCA	1,1,1-TCA; 1,1-DCA; 1,1,2-TCA→ VC		(17, 20, 22, 26, 35)
AcdA (*Dehalobacter* sp. SAD)	F	F	Y	F	W	CF	1,1,1-TCA; 1,1-DCA; 1,1,2-TCA→ VC; 1,1,2-TCA→ 1,2-DCA		(20, 23)
CtrA (*Desulfitobacterium* sp. PR)	F	F	C	L	W	CF; 1,1,1-TCA; 1,1-DCA^*a*^; 1,1,2-TCA→ 1,2-DCA	1,2-DCA^*a*^	1,1,2-TCA→ VC; DCM	(18, 36)
ThmA^*a*^ (*Dehalobacter* sp. THM)	F	F	C	L	W	CF; 1,1,1-TCA; BF		1,1-DCA; 1,1,2-TCA	(19)
RdhA 8M^*a*^*^,^^b^* (*Dehalobacter* sp. 8M)	F	F	F	F	W	CF; 1,1,2-TCA→ 1,2-DCA	1,1,1-TCA; 1,1-DCA; 1,1,2-TCA→ VC; BF		(21, 37)

^*a*^ Activity level based on culture activity instead of direct enzyme activity.

^*b*^ Published as RdhA D8M_v2_40029 (TmrA), referred to here as RdhA 8M to avoid confusion.

^*c*^ Full, incomplete, or no reduction refers to culture studies. The activity level percentage is relative to the enzyme’s highest measured activity. BF, bromoform; CF, chloroform; DCA, dichloroethane; TCA, trichloroethane; VC, vinyl chloride.

We conducted site-directed mutagenesis with all five residues of interest switched to those of the opposite enzyme, that is, CfrA was mutated to have the same five residues of DcrA and vice versa. Effectively, mutants CfrA-5M and DcrA-5M have been transplanted with the active site of the other, while all other structural differences remained untouched. These mutants were used to determine if the substrate binding site is the sole determinant of the wild-type enzymes’ specificity. The roles of specific residues and their interactions were further analyzed in CfrA with subsets of the original five mutations. These included CfrA-3M—a variant targeting the three aliphatic residues in CfrA—and several double mutants and point mutants (all described in Table 3). The double and point mutants are not exhaustive but were produced to probe specific interactions based on results from previous mutants. The wild-type enzyme and mutants were all expressed in *E. coli* and extracted using nickel-affinity chromatography. The purity of the enzymes ranged due to variations in solubility and expression levels, so the protein concentrations were adjusted to account for purity (all protein values are in Table S5 in the accompanying Excel and Supplemental Text S2). Generally, DcrA and its mutants had low solubility and purity; thus, the mutagenesis studies were primarily carried out using CfrA variants which will be the only ones discussed here. Information on the DcrA mutants can be found in Supplemental Text S5.

**TABLE 3:** Enzyme and plasmid descriptions for RDase mutants discussed

Enzyme expressed	Plasmid	Description
CfrA	*p15TVL-cfrA*	Wild-type CfrA
DcrA	*p15TVL-dcrA*	Wild-type DcrA
CfrA-5M	*p15TVL-cfrA-5M*	CfrA with mutations: Y80W, F125W, C260Y, L388F, and M391W
DcrA-5M	*p15TVL-dcrA-5M*	DcrA with mutations: W80Y, W125F, Y260C, F288L, and W391M
CfrA-3M	*p15TVL-cfrA-3M*	CfrA with mutations: C260Y, L388F, and M391W
CfrA-Y80W-F125W	*pET21-cfrA-Y80W-F125W*	CfrA with mutations: Y80W and F125W
CfrA-Y80W-Y256F	*p15TVL-cfrA-Y80W-Y256F*	CfrA with mutations: Y80W and Y256F
CfrA-Y80W-C260Y	*p15TVL-cfrA-Y80W-C260Y*	CfrA with mutations: Y80W and C260Y
CfrA-F125W	*p15TVL-cfrA-F125W*	CfrA with F125W mutation
CfrA-Y80W	*p15TVL-cfrA-Y80W*	CfrA with Y80W mutation
CfrA-Y256F	*p15TVL-cfrA-Y256F*	CfrA with Y256F mutation
CfrA-C260Y	*p15TVL-cfrA-C260Y*	CfrA with C260Y mutation

### Mutants shift substrate specificity

The activity of each mutant was measured on the primary substrates CF, 1,1,1-TCA, and 1,1-DCA. An important limitation to the comparison of the absolute activity is the uncertainty of whether any of the mutations have affected the folding or substrate incorporation. Thus, the concentration of active RDase for each mutant could vary from the measured concentration. The activity of each enzyme on the three substrates can be assessed to compare the relative preferences between enzymes, but the absolute activity of each enzyme has some uncertainty.

**Fig 2:**
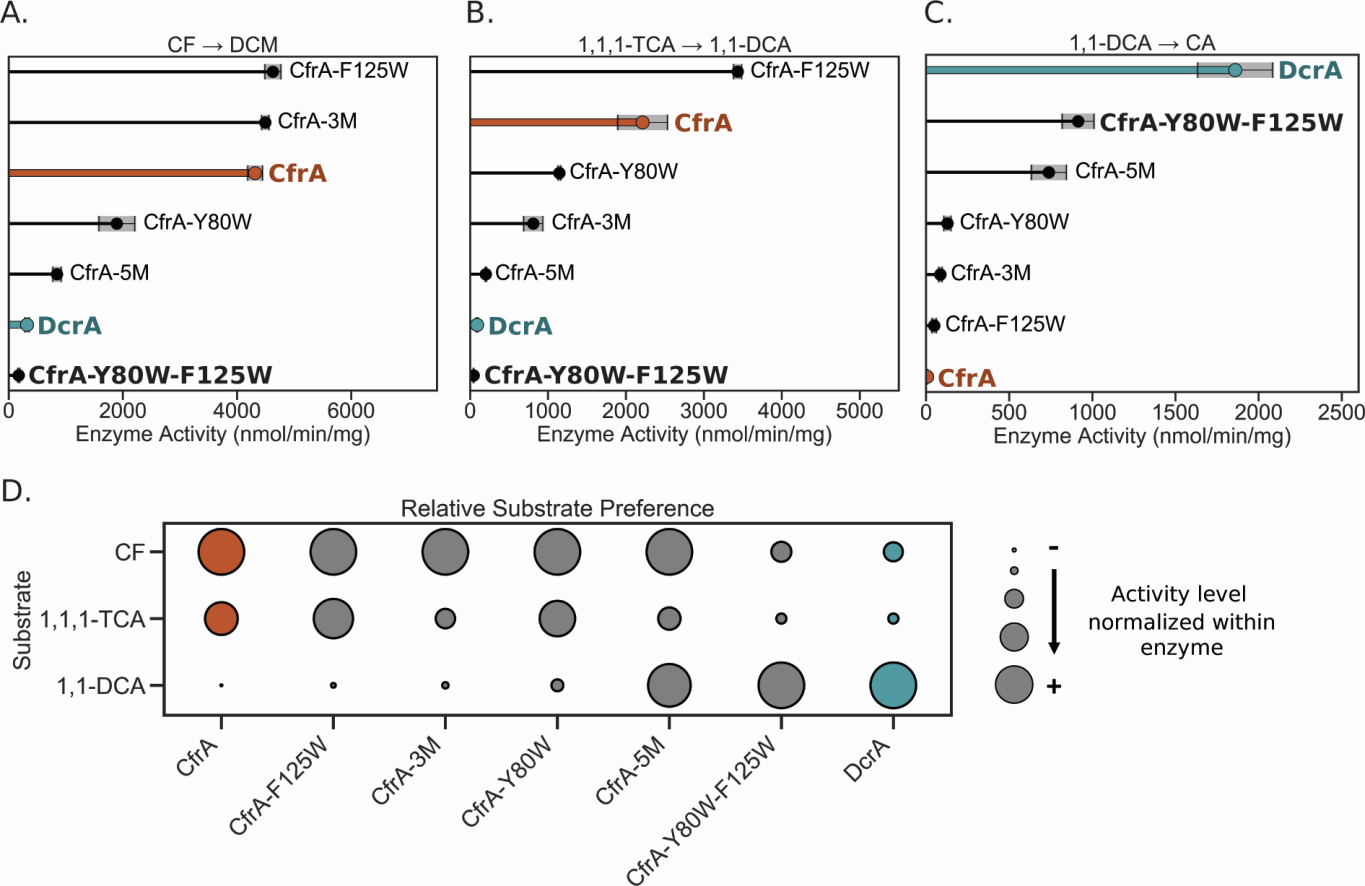
Dechlorination activity of CfrA, DcrA, and CfrA mutants on (**A**) chloroform (CF), (**B**) 1,1,1-trichloroethane (TCA), and (**C**) 1,1-dichloroethane (DCA) after 1 h. CfrA activity is highlighted in orange and DcrA in blue. Gray shading indicates the standard deviation between samples (*n* = 3 and *n* = 4 for CfrA and DcrA). (**D**) The activity of each enzyme normalized to the substrate they have the highest activity on to show their relative preferences, larger circles indicate higher relative activity levels. No products for 1,1,1-TCA and 1,1-DCA were detected in the negative controls. Only trace amounts—less than 0.2% of the average DCM released from active RDases—were detected in the negative controls with CF (Table S6). CA, chloroethane; DCM, dichloromethane.

The CfrA-5M mutant did indeed display DcrA-like behavior with diminished activity on CF and 1,1,1-TCA compared to wild-type CfrA, and new activity of 780 ± 40 nmol/min/mg on 1,1-DCA (Fig. 2A through C). However, unlike DcrA, CfrA-5M still dechlorinates CF slightly more quickly than 1,1-DCA (only 60 nmol/min/mg faster) signifying that the substrate preference is not as biased toward 1,1-DCA as it is with DcrA. Figure 2D depicts the substrate profile of each enzyme variant, where activity is normalized to the substrate with the highest activity for that enzyme (see Materials and Methods for more details). These findings affirm that the amino acids targeted with the active site transplant do influence the substrate selectivity, but differing peripheral residues may also contribute to enhanced activity. While this study focuses on active site disparities, potential additional sources of differentiation will be explored later. Here, we continue to dissect the role that these five residues play in the gain of activity on 1,1-DCA.

After confirming that transplanting the active site of DcrA into CfrA caused an appreciable gain in activity on 1,1-DCA by CfrA-5M, we sought to determine whether this change is due to the role of an individual residue or a compounding effect by several residues. Structural analysis of the five targeted residues in the wild-type RDases shows that DcrA has a more compact substrate binding pocket than CfrA (Fig. 1C and D). DcrA has five aromatic residues, three of which are tryptophan, that adds steric bulk and may help to stabilize 1,1-DCA binding but consequently prevent larger substrates like CF from binding efficiently. CfrA, on the other hand, has only two aromatic residues and three small aliphatic residues (cysteine, leucine, and methionine) that may create space to accommodate the trichlorinated substrates. The compact active sites of PceA_D2_ and DcaA were suggested to be essential for the correct positioning of the substrates—mutations that widened the active sites were disruptive to chloroethene dehalogenation (31). To narrow down the contributions of different amino acids, we first targeted CfrA’s aliphatic residues Cys260, Leu388, and Met391—all aromatic in DcrA—in the subsequent mutant CfrA-3M.

Despite CfrA-3M containing the most divergent mutations between CfrA, DcrA, and the rest of OG 97, the activity of CfrA-3M was not very different from the wild-type CfrA (Fig. 2). The activity of CfrA-3M on CF was unchanged but its activity on 1,1,1-TCA decreased by half, suggesting some steric interference with the additional methyl group on the substrate. There was a 10-fold increase in activity on 1,1-DCA; however, this is marginal compared to the activity on CF. Given these results, we chose to target the two remaining residues, Tyr80 and Phe125, to assess their role in activity on 1,1-DCA.

Both single and double mutants targeting Tyr80 and Phe125 were assayed for activity against CF, 1,1,1-TCA, and 1,1-DCA. The double mutant CfrA-Y80W-F125W exhibited the most profound change in activity shifting substrate preference to be more similar to that of DcrA, which was unexpected. These two changes in CfrA-Y80W-F125W caused an almost complete loss in activity on CF and 1,1,1-TCA and display how finely tuned substrate selection can be in RDases. Although CfrA-Y80W-F125W gained a 130-fold increase in activity on 1,1-DCA, its activity was still only half that of DcrA (Fig. 2C). One explanation may be that CfrA-Y80W-F125W has higher proportions of inactive RDase which would cause lower observed activity. Another is if there are other fundamental differences in the reduction of each substrate for which CfrA and DcrA have become specialized.

1,1-DCA has a lower reduction potential (*E*_0_′ = 397 mV) than CF (560 mV) and 1,1,1-TCA (561 mV) (38), so there is less of a driving force for the reduction of 1,1-DCA if the same electron donor is used. To make this reaction more energetically favorable, DcrA may have peripheral modifications that alter the redox potential of the cobalamin or [4Fe-4S] cofactors. In particular, [4Fe-4S]-dependent enzymes have been shown to use the amino acids that surround the clusters to tune the cluster redox potential for optimal activity (39–41). None of the five residues explored in the active site are predicted to have direct interactions with the cofactors in the protein models. However, Tyr246 (residue 260 equivalent) in PceA_S_ does have direct polar interactions with the cobalamin (3); the OG 97 models may not capture this interaction. All residues predicted to have polar interactions with the cobalamin are conserved between CfrA and DcrA; only residue 373 varies within OG 97 (either a glutamine or histidine; Supplemental Text S1). We explored some potential mutants targeting residues near the cofactors in DcrA, but these yielded limited changes in activity (discussed in Supplemental Text S5).

To further narrow down the residue roles, the point mutants CfrA-F125W and CfrA-Y80W were tested. Interestingly, CfrA-F125W produced a 1.5-fold increase in 1,1,1-TCA turnover, which could be beneficial for remediation if this variant exists naturally (Fig. 2). Conversely, CfrA-Y80W resulted in diminished activity on both CF and 1,1,1-TCA, resulting in approximately half the wild-type activity. It is clear that the introduction of Trp80 has a more adverse effect on CfrA’s activity on trichlorinated substrates. Neither mutant showed increases in 1,1-DCA reduction to the level of CfrA-Y80W-F125W, indicating that these residues must work cooperatively to produce this activity. This further supports the idea that smaller substrates require a tighter active site to be held in the ideal conformation.

### Mutants change mechanism preference

**Fig 3:**
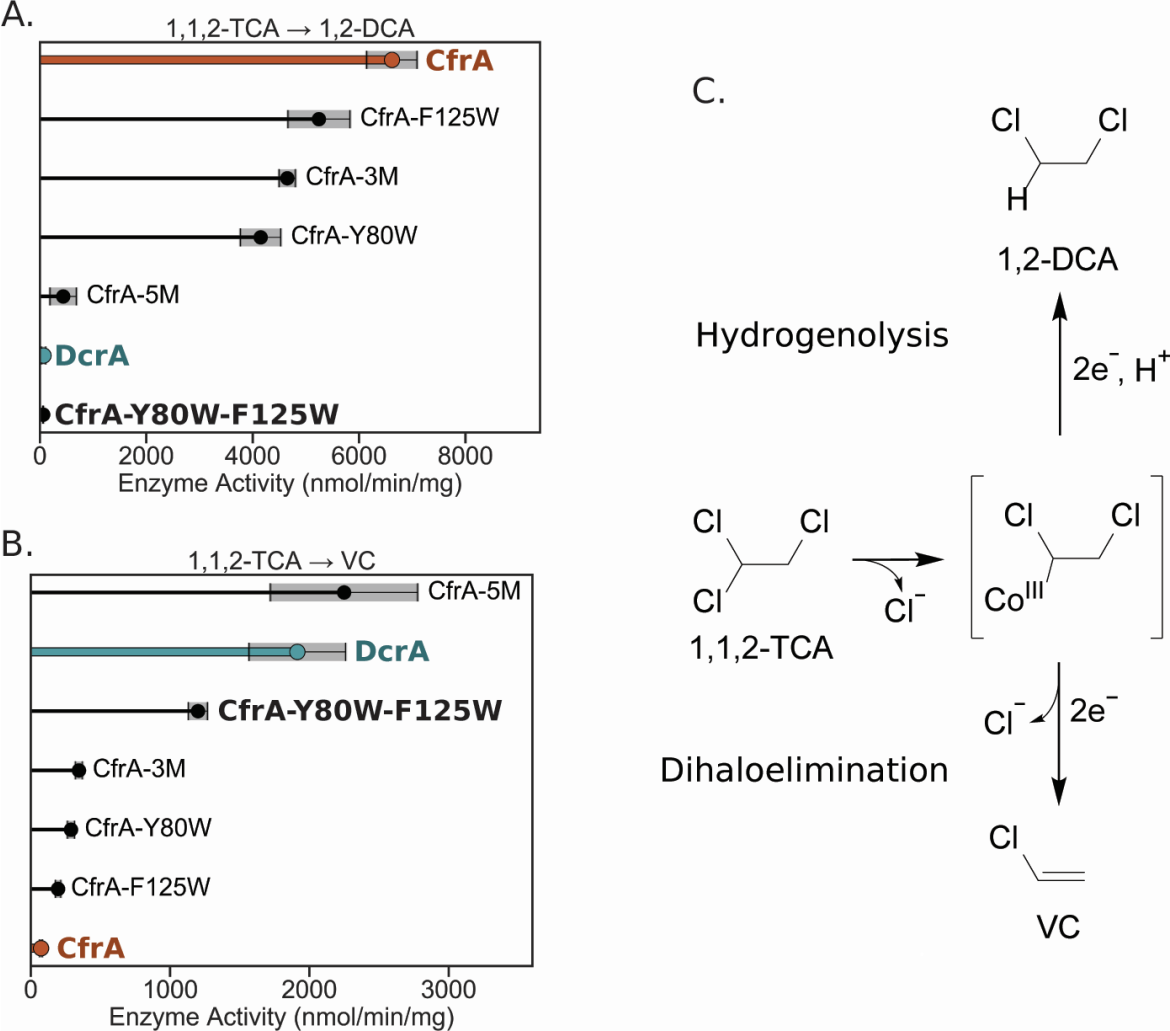
Dechlorination activity of CfrA, DcrA, and mutants on 1,1,2-trichloroethane (TCA) to produce (**A**) 1,2-dichloroethane (DCA) or (**B**) vinyl chloride (VC) after 1 h. (**C**) The two reaction pathways that 1,1,2-TCA can undergo, brackets represent the reaction intermediate based on the reaction mechanism proposed in reference 42. CfrA activity is highlighted in orange and DcrA in blue. Gray shading indicates the standard deviation between samples (*n* = 3 and *n* = 4 for CfrA and DcrA). No 1,2-DCA was detected in the negative controls, VC was produced in the reduced cobalamin controls at a rate <1% of an active RDase (Table S6).

In addition to the changes in substrate selectivity observed by CfrA-5M and CfrA-Y80W-F125W, we also observed a shift in the transformation pathway of 1,1,2-TCA. Members of OG 97 can carry out both the hydrogenolysis of 1,1,2-TCA to 1,2-DCA or the dihaloelimination to VC (Fig. 3C), but the ratio of products varies depending on the enzyme (17, 26, 36, 37). CfrA and DcrA are both on the extreme ends of either mechanism, with each producing almost homogenous products of either 1,2-DCA by CfrA or VC by DcrA (Fig. 3).

CfrA-5M and CfrA-Y80W-F125W both shifted to produce a majority of VC from 1,1,2-TCA, akin to DcrA. Once again, the CfrA-3M, CfrA-Y80W, and CfrA-F125W mutants show similar reaction profiles to CfrA, with a high preference for hydrogenolysis (>90% 1,2 DCA product, Fig. 3). The dihaloelimination reaction of 1,1,2-TCA is thermodynamically favoured with a reduction potential 201 mV higher than the hydrogenolysis reaction (38, 43). While the RDase mechanism is still under dispute, CfrA has been suggested to perform an S_N_2 reaction mechanism where a cobalt-alkyl adduct is formed (42). The formation of this adduct could be the first step for both 1,1,2-TCA transformation pathways (Fig. 3C). Given the greater driving force for reducing 1,1,2-TCA to VC, CfrA must create interactions with the substrate that enable the hydrogenolysis pathway. The next step of protonation or elimination could be dictated by surrounding residues acting as proton donors or stabilizing the β chlorine to prevent elimination. However, given that the point mutants do not have an appreciable shift toward the elimination reaction, CfrA most likely uses a network of interactions to stabilize its substrates. When these interactions are disrupted, the enzyme will revert to the easier elimination reaction as seen by CfrA-5M and CfrA-Y80W-F125W.

When considering multiple dichlorination pathways with regards to remediation, the transformation of 1,1,2-TCA to 1,2-DCA is the preferred pathway not only because VC is ranked higher and more toxic on the Substance Priority List by the Agency for Toxic Substances and Disease Registry (1), but also because VC strongly inhibits CF and 1,1,1-TCA dechlorination by CfrA (44). Understanding how structural variants affect the substrates and products of RDases is crucial for predicting outcomes in bioremediation processes. However, as we have demonstrated here, this is not a trivial task.

### Predicted structural interactions

Interactions between residues and their substrates, as well as inter-residue interactions, are highly complex and often challenging to predict, especially when relying on static structural models. By employing protein docking and visualizing the active site, we can make informed predictions about the interactions that could be disrupted by mutagenesis, thereby leading to changes in activity. However, it is important to consider the limitations of current structure prediction tools, particularly regarding the precise rotamers and locations of side chains within the structure. Thus, we will be discussing our general observations of potential structural differences based on the chemical characteristics of the mutated residues. Obtaining crystal structures of the RDases would enable more accurate observations, but thus far our preliminary attempts to crystalize OG 97 RDases have been unsuccessful.

**Fig 4:**
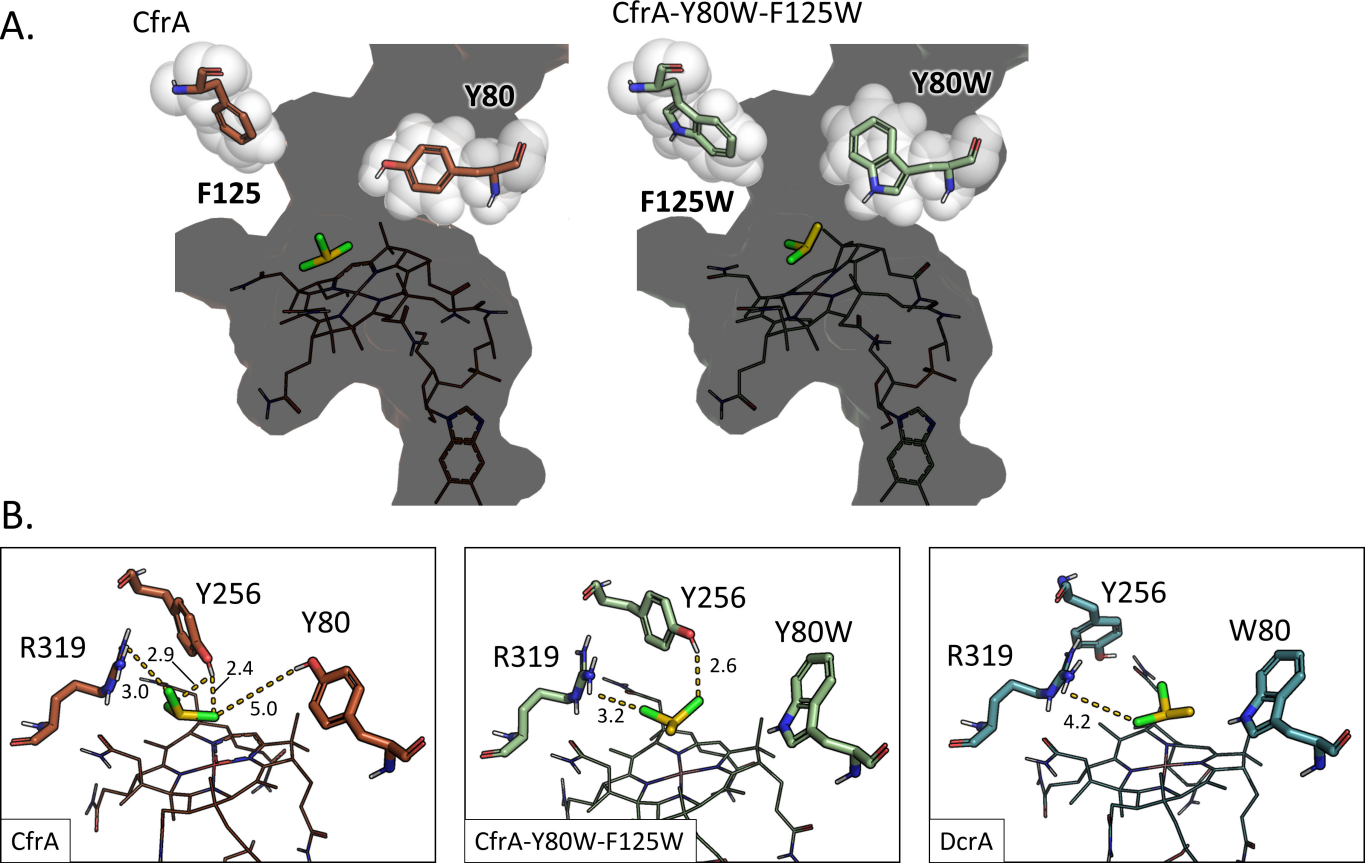
Protein structure models of CfrA, CfrA-Y80W-F125W, and DcrA show the possible interactions made by residues 80 and 125. (**A**) The positioning of residues 80 and 125 in the substrate access channel of CfrA (left; orange) and CfrA-Y80W-F125W (right; green). (**B**) Potential hydrogen-bond donors in the active sites of CfrA (orange), CfrA-Y80W-F125W (green), and DcrA (blue). Distances of atoms in Å are labeled on dashed lines. All models are docked with their preferred substrate (yellow): CfrA with chloroform and CfrA-Y80W-F125W and DcrA with 1,1-dichloroethane.

The two major differences between DcrA and CfrA are the presence of more aromatic residues in DcrA adding steric bulk and the positioning of residues that can act as hydrogen bond donors. The mutations made in CfrA-5M were all from smaller amino acids to larger aromatic residues. These residues not only take up space but can also provide hydrophobic interactions with the substrates. Yet, we observed that only two of those mutations were necessary to achieve activity similar to DcrA. Residues Tyr80 and Phe125 line the substrate access channel and mouth of the active site in the CfrA model (Fig. 4A). Mutating both residues to tryptophan could have narrowed the channel—the narrowest point between these residues is 8.4 and 7.6 Å in the CfrA and CfrA-Y80W-F125W models, respectively. Both CF and 1,1,1-TCA bear three chlorine atoms on one carbon. Since chlorine has a much larger atomic radius than hydrogen, the widest point of these substrates will be larger than 1,1-DCA or 1,1,2-TCA. The steric restriction of the channel may occlude the binding of these larger substrates. In the mutagenesis of *D. dichloroeliminans* DcaA, the opposite effect was observed, where decreasing the residue size at the equivalent of Phe125 (Trp432 in DcaA) decreased the activity on all substrates (31). The data reported here, however, suggest that residue 80 has a greater impact on CfrA’s activity. CfrA-Y80W consistently has lower activity than CfrA, but CfrA-F125W has increased activity on 1,1,1-TCA.

Another differentiator between CfrA and CfrA-Y80W-F125W is the potential for hydrogen (or halogen) bonding by Tyr80 and other neighboring residues. Tyrosine forms stronger hydrogen bonds and has a higher propensity to form halogen bonds than tryptophan (45–47). The hydroxyl group has the ability to create a strong bond with the substrate chlorines, which may help direct the substrate into a reactive orientation. For the chloroalkane RDases to perform S_N_2 substitution reactions, they may need to coordinate trichlorinated substrates effectively to overcome the steric barrier for the nucleophilic cobalt to reach the electrophilic carbon. Therefore, the number and type of stabilizing interactions CfrA and DcrA make with their substrates and cofactors could be a factor in their selectivity.

In the CfrA active site, there are three potential hydrogen bond donors—Tyr80, Tyr256, and Arg319 (Fig. 4B). Arg319 is predicted to be the proton donor involved in catalysis and is structurally conserved among all OG 97 members (32). Tyr256 is conserved in all OG 97 sequences, but its predicted positioning in the protein structures varies (Fig. S9). When the models were docked with their substrates, CfrA was the only one that had Tyr256 interacting with the substrates (Table S3). While we cannot be certain of this residue’s orientation in the models, we can look at how the surrounding residues may interact with it.

**Fig 5:**
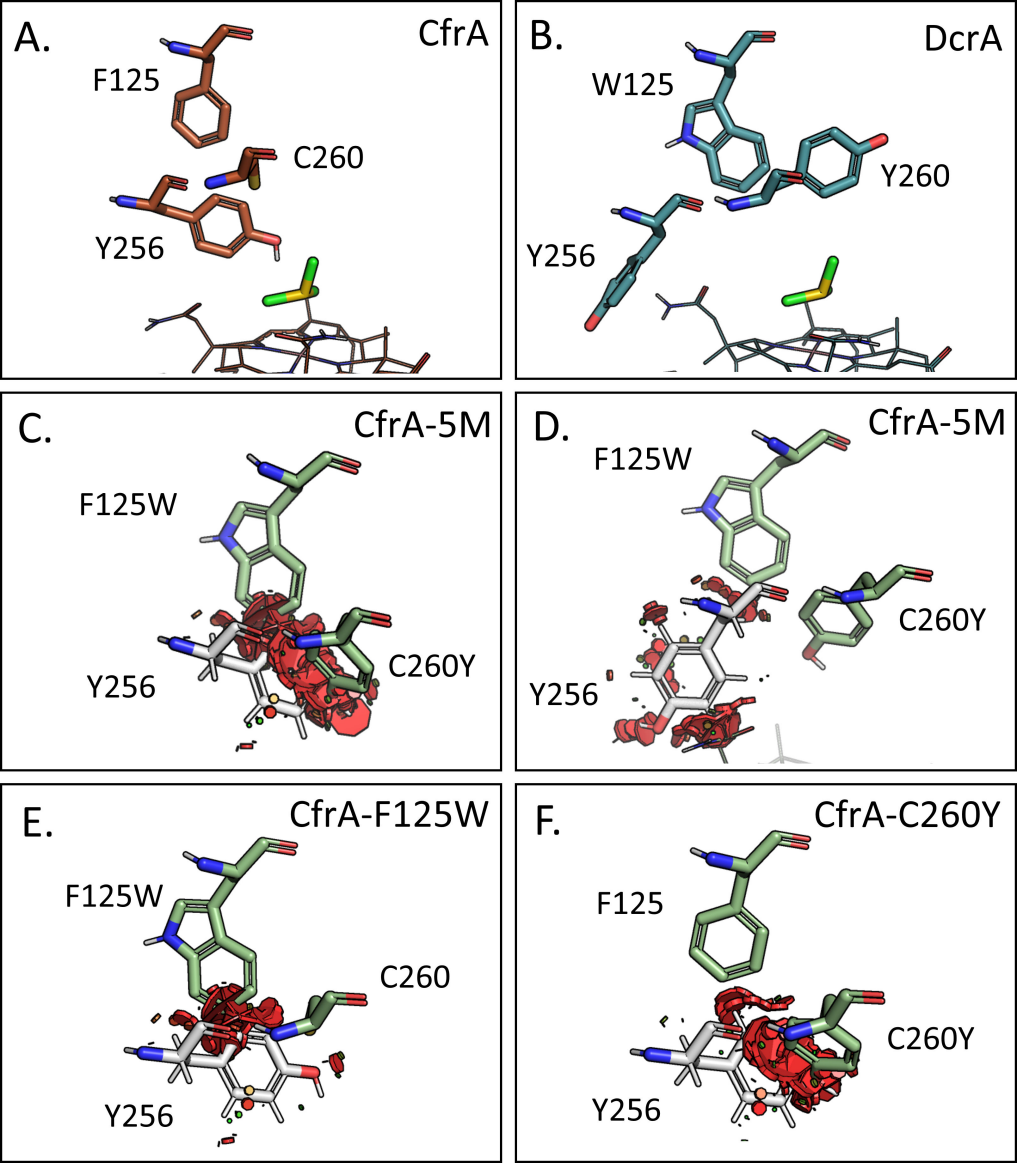
Protein structure models of (**A**) CfrA and (**B**) DcrA show the predicted positioning of Tyr256 in the active site; the docked substrate is shown in yellow and cobalamin is shown as a wire figure. CfrA-5M model with the steric clash resulting from the (**C**) Tyr256 (white) rotamer pointing toward the active site and (**D**) the rotamer pointing out of the active site. Tyr256 pointing toward the active site and the resulting clash with each of the (**E**) F125W and (**F**) C260Y mutations individually. Red disks indicate steric clash where Tyr256 overlaps with the van der Waals radius of atoms in neighboring residues; only residues at positions 125 and 260 are shown as green sticks.

In Fig. 5, the relative positions of Tyr256 are shown with varying residues 125 and 260 in CfrA and DcrA. DcrA has bulky aromatics at both positions and thus, steric clash may prevent Tyr256 from rotating into the active site. CfrA does not have these restraints. To illustrate the potential steric clash and assess how our designed mutations may affect the Tyr256 positioning, we analyzed the different Tyr256 rotamers in the CfrA-5M model using the PyMOL Wizard mutagenesis tool. Using this tool, we can assess possible rotamers of Tyr256 and how likely they are to clash with neighboring residues. In Fig. 5, the steric clash is shown with red disks, for which the size and density of the disks show the relative degree of overlap between the van der Waals radii of the atoms in the residues—that is, greater overlap and steric clash displays more red disks in the images and would correspond to a less favorable conformation. In the CfrA-5M model active site, there is less room for Tyr256 and high steric clash is predicted if it faces toward the substrate binding location (Fig. 5C and D). Both F125W and C260Y contribute to this clash, as we still see steric overlap when the mutations are visualized individually (Fig. 5E and F), but the preferred rotamers for the single mutants are not as obvious. The interactions between the introduced mutations and other important active site residues could also contribute to the activity changes we see in the CfrA mutants (Table S4).

It is unclear whether Tyr256 is important in substrate selectivity in OG 97, but the equivalent Thr294 in DcaA was shown to have a great impact on substrate selectivity (31). The T294V mutant of DcaA enhanced preference for dehalogenation of trichloroethane over PCE and reduced dihaloelimination activity on 1,1,2-TCA and 1,2-DCA (31). The authors attributed this change to a slight increase in residue size, suggesting that dihaloelimination may be favoured when there is more active site space at this position. This could mirror what we observe between CfrA, DcrA, and the mutants. Another possibility is the potential stabilizing hydrogen bonding between Tyr256 and the substrate.

Additionally, these residues could influence cobamide bonding. Arg319 is predicted to have a polar interaction with the cobamide cofactor, Tyr256, and residue 260 (Fig. S10). These interactions were also present in the crystal structures of PceA_S_ and PceA_D1_ (3, 28). It is possible that the cobamide interactions affect the nucleophilic cobalt center, so surrounding residues that alter the nature of these interactions could impact the enzyme activity. Similarly, the backbone of residue 80 has an interaction conserved residue Glu78 which hydrogen bonds with the cobamide; the mutations may affect the length and angle of this interaction (Fig. S11). We produced several more double and single to delve into these possible interactions.

### Influence of individual residues

The second round of mutants was produced to assess the role of Tyr256 and Cys260, particularly in 1,1,2-TCA transformation. To determine if the hydrogen bonding capability of Tyr256 is important to activity, it was mutated to phenylalanine. This change still maintains the bulk and hydrophobicity provided by the aromatic ring, with only a small decrease in size, but removes the hydrogen bonding hydroxyl. C260Y was targeted due to its proximity to Tyr256, as it may clash with Tyr256 and push it out of the active site (Fig. 5F). Further, residue 260 is highly variable within OG 97, and Tyr260 is present in DcrA and AcdA, which both perform the dihaloelimination reaction more readily than CfrA (20, 26). Both Y256F and C260Y were paired with Y80W in double mutants of CfrA, because Y80W appeared to have more influence over activity than F125W alone.

**Fig 6:**
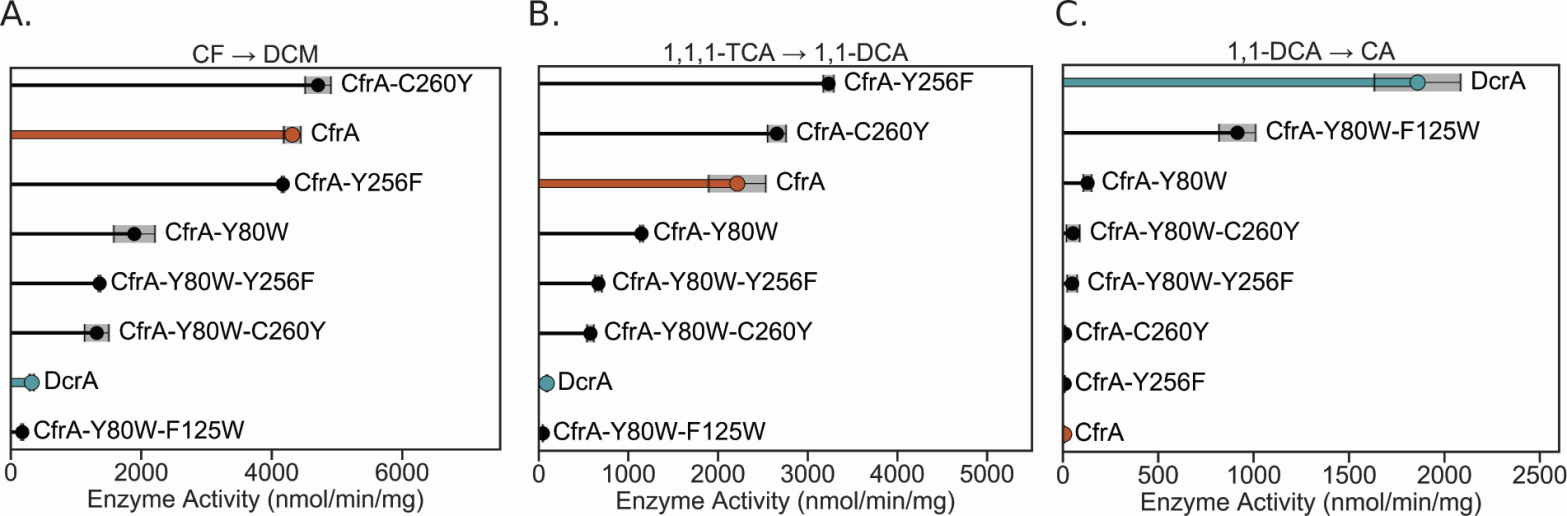
Dechlorination activity of CfrA, DcrA, and select mutants on (**A**) chloroform (CF), (**B**) 1,1,1-trichloroethane (TCA), and (**C**) 1,1-dichloroethane (DCA) after 1 h. CfrA activity is highlighted in orange and DcrA in blue. Gray shading indicates the standard deviation between samples (*n* = 3 and *n* = 4 for CfrA and DcrA). CA, chloroethane; DCM, dichloromethane.

**Fig 7:**
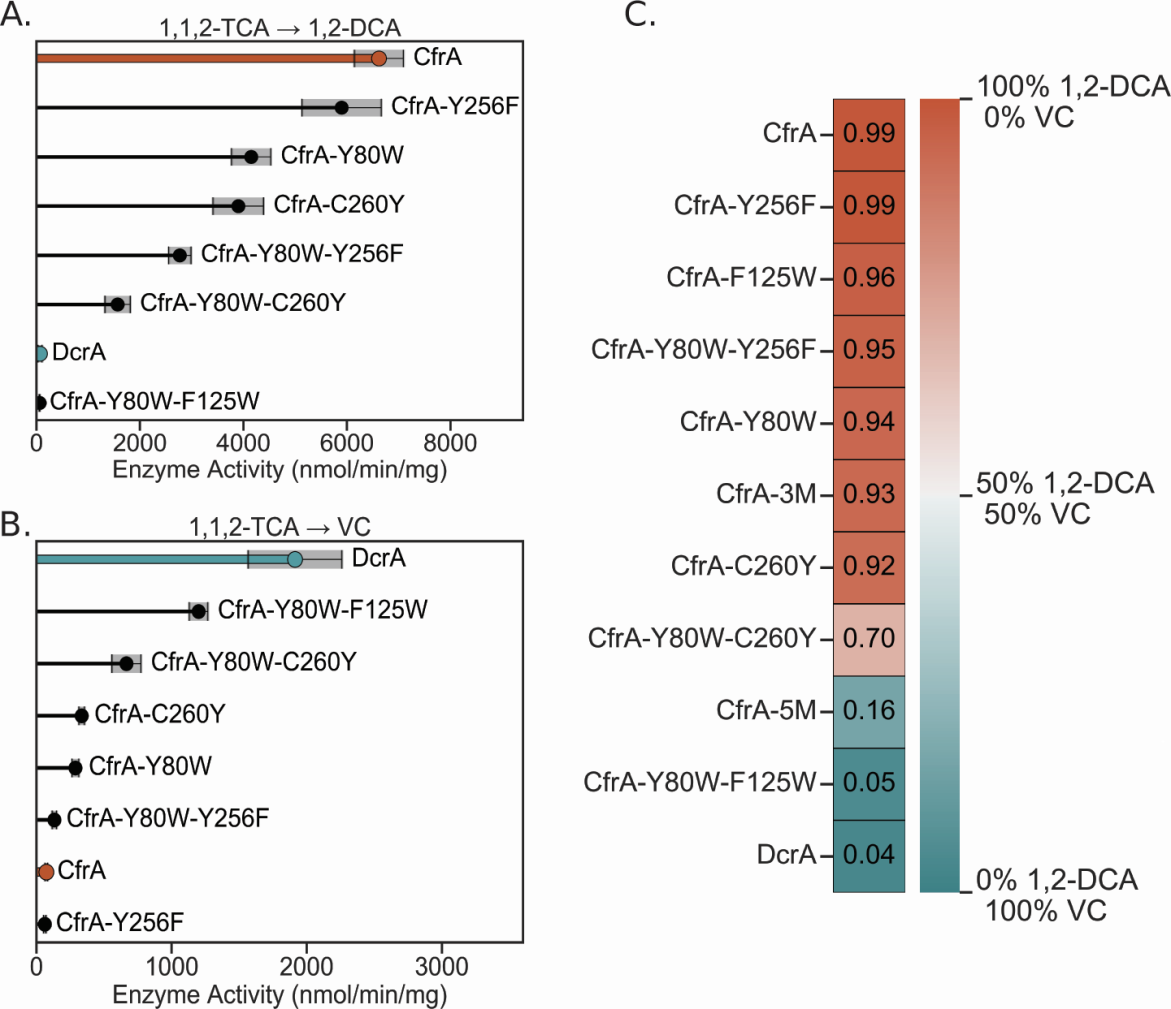
Dechlorination activity of CfrA, DcrA, and select mutants on 1,1,2-trichloroethane (TCA) to produce (**A**) 1,2-dichloroethane (DCA) or (**B**) vinyl chloride (VC) after 1 h. (**C**) The proportion of the dechlorination product that went to 1,2-DCA (more orange) and VC (more blue); the number in the square indicates the ratio of 1,2-DCA product. CfrA activity is highlighted in red, DcrA in blue, and CfrA-Y80W-F125W in green. Gray shading indicates the standard deviation between samples (*n* = 3 and *n* = 4 for CfrA and DcrA).

The single mutants (Y256F and C260Y) did not hold much control over the substrate preferences of CfrA (Fig. 6). It is unlikely that Tyr256 participates in hydrogen bonding for substrate selection because it maintained the same activity level and preferences as CfrA (Fig. 6 and 7). Yet, when paired with Y80W, the CfrA-Y80W-Y256F double mutant lost 60–70% of its activity on CF, 1,1,1-TCA and 1,1,2-TCA. A similar trend was seen with CfrA-C260Y, where it retained activity on CF and 1,1,1-TCA but lost 70% of the activity when paired with Y80W. These decreases could be caused by a true reduction in substrate turnover, or they could be caused by changes in the folding and cofactor incorporation, resulting in a lower concentration of active RDase.

Neither CfrA-Y80W-Y256F nor CfrA-Y80W-C260Y could dechlorinate 1,1-DCA. This supports the idea that increasing the bulk at the substrate access channel, as seen in CfrA-Y80W-F125W, improves activity on smaller and less halogenated substrates. Perhaps narrowing the substrate access channel prevents the substrate from leaving before catalysis. Kinetic analysis to assess how residues 80 and 125 impact the *K*_M_ on 1,1-DCA would be needed to further support this hypothesis.

While the change in Tyr256 did not affect the transformation pathway of 1,1,2-TCA, the C260Y mutation did have a slight effect, especially when paired with Y80W. CfrA-Y80W-C260Y retained less than 25% of the wild-type hydrogenolysis activity on 1,1,2-TCA but increased the dihaloelimination reaction 10-fold (Fig. 7). On its own, the C260Y mutation shifted the percentage of VC in the product from 1% (wild-type) to 8%, and the double mutant CfrA-Y80W-C260Y increased the VC% in the product to 30% (Fig. 7C). The large increase in VC production from the double mutant further indicates that there are multiple interactions by CfrA to suppress dihaloelimination. The effect of C260Y may be due to its bulk preventing Tyr256 from entering the active site, and the open space increases the dihaloelimination activity, as we have discussed. Tyr80, on the other hand, could be involved in changing the orientation of 1,1,2-TCA as it enters the active site and could also provide stabilization through hydrogen bonding. TmrA and AcdA have have a non-hydrogen-bonding Phe80 and bulky aromatic residues at 260 which could be why they produce mixtures of VC and 1,2-DCA (Table 2; Fig. S3). On the contrary, CtrA also has Phe80 but produces only trace VC; it does have Cys260 similar to CfrA. The interactions to determine substrate specificity do not appear to be universal within OG 97, but the results here provide some insight into trends in residue interactions and further highlight the need for experimental structures. Additionally, assessing the role of enzyme-cofactor interactions—for example, comparing the cofactor reduction potential in the mutants—could illuminate other means of activity differentiation.

### Significance and conclusions

By manipulating the amino acid composition of CfrA’s active site, we have found residues that are highly influential in selecting larger trichlorinated substrates versus dichlorinated, and those that can direct the reaction mechanism of 1,1,2-TCA toward one product over another. The two mutations that completely changed CfrA’s substrate specificity were Y80W and F125W. These residues line the entrance to the active site from the substrate access channel and may act as a filter to block larger trichlorinated substrates while also stabilizing 1,1-DCA for catalysis. This theory could be tested by assessing activity on brominated alkanes, which are bigger but act as better leaving groups for dehalogenation. Corresponding residues have been reported or suggested to be important in determining the activity of PceA_S_, PceA_D2_, and DcaA due to their proximity to the active site (3, 29–31). Similar to DcaA, we observed that bulkier residues in the active site have an influence on the size of the preferred substrates.

Further, we have observed that the active site residues in OG 97 produce compounding effects on enzyme activity and substrate selectivity. No single point mutation increased 1,1-DCA activity; however, residue 80 seems to have a greater influence in selecting trichlorinated substrates. When several active site mutations were combined, we saw the greatest increase in non-wild-type activity, suggesting synergistic contributions to function.

By analyzing the diverging evolution of CfrA and DcrA that specialized them toward separate substrates, and comparing this to chloroethene-reducing RDases, we have identified several residues that could be hotspots for natural evolution and protein engineering. Changes in residues 80, 125, and 256 (Phe38, Trp96, and Thr242 in PceA_S_), both in the side chain and potentially the positioning of the residues, appear to have large impacts on the preferred substrate size and the tendency to perform dihaloelimination (31). The effect of the residue size is consistent between the CfrA and DcaA mutagenesis results. Adding bulkier residues, particularly at the mouth of the active site, favors smaller substrates. Introducing more space into the active site has been detrimental to the activity on small substrates, but there is no clear consensus on the active site space and inclination for dihaloelimination (31). Variations between residues might also alter a network of conserved residues, so surrounding residues should also be considered when introducing mutations. Crystal structures and molecular dynamics analysis of CfrA and DcrA would help confirm these observations.

It is unlikely that the active site is the only source of substrate differentiation, and other areas that should be further assessed are residues at the opening and along the substrate access channel. As shown in Fig. 1 the natural variations between CfrA and DcrA are clustered around the substrate channel; key differences here could be why the CfrA mutants do not reach the same level of activity on 1,1-DCA as the wild-type DcrA. Another impact on enzyme activity—which has yet to be explored in RDases—is the environment surrounding the iron-sulfur clusters and cobalamin. It is known that the protein structure impacts the reduction potential of redox-active cofactors; factors such as solvation, and hydrogen-bonding or ionic residues surrounding the iron-sulfur clusters can drastically change their reduction potential and therefore, the enzyme’s activity (41, 48). While none of the residues studied here were predicted to have direct interactions with the cofactors, they could have secondary impacts on important cofactor-residue interactions resulting in a change in activity.

These findings suggest that CfrA has specialized residues to accommodate 1,1,1-TCA as a substrate. The mutants, particularly CfrA-3M, showed varied changes in activity levels between CF and 1,1,1-TCA. Adding steric bulk in the active site reduced 1,1,1-TCA activity more than CF, while changes in the substrate access channel impacted both similarly. Kinetic analysis indicates that CfrA has a higher *K*_M_ with 1,1,1-TCA (44, 49, 50), meaning lower affinity. This difference in *K*_M_ and sensitivity to active site size aligns with CfrA isotope fractionation data showing increased masking (less fractionation) of 1,1,1-TCA compared to CF (51–53). Masking occurs when a non-fractionating slow step governs the rate rather than the fractionating transformation step. CfrA has no masking with CF as it has fractionation patterns similar to cobalamin (34, 53). The considerable effect that minor active site differences had on 1,1,1-TCA activity suggests that the masking likely results from differences in substrate binding. If 1,1,1-TCA has a tighter fit to bind the active site, this could repress the reaction rate without causing fractionation leading to masking effects. However, further isotope analyses and mechanistic studies are needed to confirm.

The findings presented here underscore the challenge of accurately predicting RDase activity based solely on sequence information. One cannot presume identical activity between RDases, even if they share high sequence similarity. Additionally, they contribute to our understanding of the factors influencing substrate selectivity in RDases and hold promise for guiding future rational engineering efforts to accommodate novel substrates.

## MATERIALS AND METHODS

### Chemicals and other materials

Unless otherwise stated, all chemicals, BugBuster, and filter tubes were purchased from Millipore Sigma (Burlington, MA, USA). All growth media, antibiotics, and induction agents were purchased from BioShop Canada Inc. (Burlington, ON, Canada). PCR mix, restriction enzymes, and Gibson assembly mix were purchased from New England Biolabs Ltd. (Ipswich, MA, USA). Bradford reagent was purchased from Bio-Rad Laboratories Ltd. (Hercules, CA, USA). All gasses were supplied from Linde Canada Inc. (Mississauga, ON, Canada). A Coy vinyl anaerobic chamber (Coy Laboratory Products Inc., Grass Lake, MI, USA) was supplied with a gas mix of 10% CO_2_/10% H_2_/Bal. N_2_ (vol/vol) is used to maintain an anaerobic atmosphere, which is referred to as a glovebox throughout. Gas chromatography materials were purchased from Agilent Technologies (Santa Clara, CA, USA). All genes were confirmed by Sanger sequencing at The Centre for Applied Genomics (TCAG; SickKids, Toronto, ON, Canada).

### Sequence and structure comparison

Table 1 shows residues in CfrA and DcrA equivalent to those in PceA_S_ and DcaA described in the literature (3, 29–31). The residues were identified through a multiple sequence alignment using MAFFT v7.017 and structural analysis using PyMOL (54, 55). The amino acid sequences of all functionally characterized RDases in OG 97 (CfrA, DcrA, TmrA, AcdA, CtrA, ThmA, and RdhA D8M_v2_40029) were aligned using MAFFT v7.017 plugin in Geneious v8.1.9 with default settings (54). Predictive protein models were made for CfrA, DcrA, TmrA, and AcdA using AlphaFold2 (56). Each non-conserved residue in the alignment was visualized in the protein models using PyMOL v2.3.4 to determine their proximity to the putative substrate binding site and their likelihood to contribute to activity differences observed between the enzymes (55). Five variable residues at positions 80, 125, 260, 388, and 391 were identified in the active site and were pursued in mutagenesis studies—selection described in Results and Discussion. Residues 80, 260, 388, and 391 were also noted as potentially important residues in the isotope fractionation study comparing TmrA, CfrA, and AcdA (34).

### Plasmid construction

Plasmids for CfrA and DcrA were constructed previously (26). Genes for the mutants CfrA-5M, CfrA-3M, and DcrA-5M were codon optimized and purchased from Twist Bioscience (San Francisco, CA, USA) and cloned into *p15TV-L* plasmid as described previously (26). Briefly, the genes had their TAT signal peptides removed from the sequences as determined by SignalP6.0 (57). The synthetic genes were inserted into the *p15TV-L* plasmid linearized with BseRI digestion using Gibson assembly.

Expression plasmids for point mutants CfrA-Y80W, CfrA-F125W, CfrA-C260Y, CfrA-Y256F, and double mutants CfrA-Y80W-C260Y and CfrA-Y80W-Y256F were constructed using the site-directed PCR mutagenesis protocol from Edelheit et al. (58). The wild-type CfrA plasmid, *p15TVL-cfrA*, was used as the template for mutagenesis for the single mutants, and the constructed *p15TVL-Y80W* plasmid was used as the template for the double mutants. Primers used to introduce the mutations are in Table S1. All assembled plasmids were confirmed by Sanger sequencing using universal T7 and T7 terminator primers. CfrA-Y80W-F125W was codon optimized and synthesized in the *pET-21* plasmid along with sequences encoding a ribosome binding site, N-terminal histidine tag, and TEV cleavage site added upstream of the gene (Twist Bioscience). Additional mutants and enzymes are described in Table S2 and their activity can be found in the supplementary material.

All expression plasmids were co-transformed with *pBAD42-BtuCEDFB* into *E. coli* BL21(DE3) *cat-araC-*P_BAD_-*suf*, Δ*iscR::kan*, Δ*himA::*Tet^R^, abbreviated to *E. coli ara-Suf* Δ*iscR* (59, 60). The *pBAD42-BtuCEDFB* plasmid, used for cobalamin uptake, was generously provided by the Booker Lab (Pennsylvania State University, PA, USA). *E. coli* BL21(DE3) *cat-araC*-P_BAD_-*suf*, *ΔiscR::kan*, *ΔhimA::*Tet^R^, used for enhanced iron-sulfur production, was generously provided by the Antony and Kiley Labs (St. Louis University School of Medicine, MO, USA; University of Wisconsin-Madison, WI, USA).

### Expression and nickel-affinity chromatography

Methods for RDase expression and enrichment by nickel-affinity chromatography have been described elsewhere (20, 26). Minor amendments were made here. Briefly, for each enzyme, an overnight starter culture was used to inoculate 1 L, 500 mL, or 250 mL of Luria Broth (LB) in 2 L, 1 L, or 500 mL media bottles, respectively, to ensure consistent liquid and headspace volumes. The cultures were grown aerobically at 37°C, 170 rpm until the OD_600_ reached 0.2–0.4, at which 3 µM hydroxycobalamin and 0.2% (wt/vol) ʟ-arabinose were supplemented to induce Btu expression. The cultures were capped and incubated again at 37°C until the OD_600_ reached ~0.6–0.8, after which the cultures were purged with N_2_ gas for 1 h/L liquid volume. The cultures were supplemented with 50 µM cysteine and 50 µM ammonium ferric citrate, and RDase expression was induced with 0.3–0.5 mM isopropyl β-ᴅ-1-thiogalactopyranoside (IPTG). Finally, the culture was incubated for 18 h at 15°C, 170 rpm.

The cultures were kept anaerobic and pelleted by centrifugation, and all subsequent steps were performed in the glovebox or sealed vessel to maintain anaerobicity. The pellet was resuspended in 5 mL of Lysis Buffer (50 mM Tris-HCl pH 7.5, 150 mM NaCl, 0.1% Triton, and 5% glycerol) per 1 g of wet cell weight; a 1 L culture would usually yield 3–4 g. Reagents 10× Bug Buster concentrate (used at 1×), 1 mM tris(2-carboxyethyl)phosphine (TCEP), 50 µg/mL leupeptin, 2 µg/mL aprotinin, 0.3 mg/mL lysozyme, 1.5 µg/mL DNase, and 10 mM MgCl_2_ (final concentrations) were all added fresh. This was incubated at room temperature shaking at 50 rpm for 20 min. The lysate was clarified by centrifugation at 30,000 × *g*, 4°C for 20 min and incubated with nickel-nitrilotriacetic acid (Ni-NTA) beads either in the column for lysate quantities ≥10 mL, or in batches for smaller lysate quantities.

The Ni-NTA beads were washed with Wash buffer (50 mM Tris-HCl pH 7.5, 150 mM NaCl, and 30 mM imidazole) until no protein could be detected with Bradford reagent. The RDases were eluted using Elution buffer (50 mM Tris-HCl pH 7.5, 150 mM NaCl, and 300 mM imidazole). The eluant was exchanged into Storage buffer (50 mM Tris-HCl pH 7.5 and 150 mM NaCl) and concentrated using a 30 kDa cutoff Millipore filter tube. The protein was stored in liquid nitrogen. The concentrate was quantified with a Bradford assay and the purity was assessed by SDS-PAGE using Image Lab v6.1 Software (Bio-Rad Laboratories Inc., 2020; Fig. S1; Table S5 in the accompanying Excel).

### Reductive dechlorination assays

The dechlorination activity of each mutant and wild-type enzyme was assessed on the substrates CF, 1,1,1-TCA, 1,1,2-TCA, and 1,1-DCA; some enzymes (CfrA, DcrA, CfrA-5M, CfrA-3M, and CfrA-Y80W-F125W) were also assessed for activity on 1,2-DCA and dichloromethane (DCM) (Fig. S4). Each enzyme assay was carried out anaerobically in 500 µL reaction volumes using 2 mL vials with 500 µL glass inserts sealed with a Teflon-coated cap such that there was no headspace in the vial. The reaction mix contained 5 mM Ti(III) citrate and 2 mM methyl viologen in 50 mM Tris-HCl pH 7.5. Substrates were added to a target concentration of 0.5 mM using saturated water stocks. Enzymes were added in a volume of 5 µL to deliver 0.2–0.6 µg of RDase (accounting for purity). The reactions were incubated at room temperature for 65 min. The reaction was stopped by transferring 400 µL into 5.6 mL HCl-acidified water (pH < 2) in an 11 mL headspace vial and crimped for analysis by gas chromatography-flame ionization detection (GC-FID).

The samples were all analyzed by using GC-FID separation on an Agilent 7890A GC instrument equipped with an Agilent GS-Q plot column (30 m length and 0.53 mm diameter). Sample vials were equilibrated to 70°C for 40 min in an Agilent G1888 autosampler, then 3 mL of the sample headspace was injected (injector set to 200°C) onto the column by a packed inlet. The flow rate was 11 mL/min of helium as the carrier gas. The oven was held at 35°C for 1.5 min, raised at a rate of 15°C/min up to 100°C, and the ramp rate was reduced to 5°C/min until the oven reached 185°C at which point it was held for 10 min. Finally, the oven was ramped up to 200°C at a rate of 20°C/min and held for 10 min. The detector was set to a temperature of 250°C. Data were analyzed using Agilent ChemStation Rev.B.04.02 SP1 software, and peak areas were converted to liquid concentration using external standard curves for each compound.

Enzyme-free and free cobalamin (0.0005 mg) negative control reactions were performed for each substrate. All reactions were done in technical triplicates; CfrA and DcrA were assayed using four replicates. The mass of the dechlorinated product was calculated from the liquid concentration and apparent activity was calculated by dividing the product mass by the incubation time and the amount of enzyme in the reaction. All data for OG 97 RDases and CfrA mutants are in Fig. S2 and S3; additional activity values on DCM and 1,2-DCA are shown in Fig. S4. All raw data for activity is reported in the accompanying Excel document in Tables S6 and S7.

### Activity normalization

The substrate preference of each enzyme was visualized using a bubble plot. For each enzyme, the activity level on substrates CF, 1,1,1-TCA, and 1,1-DCA was normalized by dividing by the highest activity observed by that particular enzyme (equation 1 in Supplemental Text S3). This normalization scaled the activities from 0 (no activity) to 1 (highest activity). The size of each bubble in the plot represents the normalized activity, allowing for a visual comparison of how each enzyme interacts with the three substrates. However, it does not allow for a direct comparison of activity levels between different enzymes. Additionally, in Text S3 a dendrogram was produced to cluster the enzymes based on activity similarity (Fig. S5). This clustering was performed by normalizing the activity of all enzymes to the activity of CfrA and DcrA depending on the substrate (see equations 2 and 3 and Text S3 for further details).

### Protein models and docking

New protein models of CfrA, DcrA, and TmrA were obtained through the AlphaFill webserver with cobalamin and [4Fe-4S] clusters incorporated into the structures (61). The models were retrieved using UniProt IDs: CfrA, K4LFB7; DcrA, J7I1Z7; and TmrA, T0I1B4. The models were relaxed using the YASARA energy reduction web server (62). Water molecules were removed from these models and they were used for docking studies with AutoDock4.2 (63). Each protein was docked with CF, 1,1,1-TCA, 1,1-DCA, and 1,1,2-TCA to proximate the binding location and residue interactions with each substrate. All AutoDock settings were kept as default, cobalt was added to the parameters file as a heteroatom, the enzymes were kept rigid with no flexible residues, and the docking grid space was located on the open face of the docked cobalamin molecule. The top docking position for each enzyme-ligand pairing was used to assess binding affinity and interacting side chains (Table S3).

The protein model of AcdA was produced using AlphaFold2 (56). The model was superimposed with the cobalamin and [4Fe-4S] clusters from the CfrA model in PyMOL and exported as a new model to incorporate the cofactors. The mutant models were created from the CfrA AlphaFill model by using the PyMOL Wizard mutagenesis tool to introduce the mutation in the most favorable rotamer. The edited models were relaxed using the YASARA energy minimization server. These were all docked with CF, 1,1,1-TCA, 1,1-DCA, and 1,1,2-TCA using the same methods as described above. Images of the undocked protein structure active sites are in Fig. S6 and S7.

The steric clash between Tyr256 rotamers and the surrounding residues was assessed using the Wizard mutagenesis tool in PyMOL. The relative distance between mutated residues was also recorded to compare which mutations introduce more strain in the model. The observed strain score and the rotamer probability are in Table S4; Fig. S8 and S9.

## SUPPLEMENTAL MATERIAL

Supplemental material aem.01501-24-s0001.pdf Additional information on mutant construction, purification, assay data, protein model and substrate docking analysis, and DcrA mutants.

Tables S5 to S7 aem.01501-24-s0002.xlsx RDase concentrations, and all raw activity data for enzymes and controls.

ASM does not own the copyrights to Supplemental Material that may be linked to, or accessed through, an article. The authors have granted ASM a non-exclusive, world-wide license to publish the Supplemental Material files. Please contact the corresponding author directly for reuse.
