# Supplementary material for: Deciphering reductive dehalogenase specificity through targeted mutagenesis of chloroalkane reductases
Source: Appl Environ Microbiol. 2025 Feb 13;91(3):e01501-24. doi: 10.1128/aem.01501-24 (PMC11921367; doi:10.1128/aem.01501-24)
Supplement: Supplemental material — Additional information on mutant construction, purification, assay data, protein model and substrate docking analysis, and DcrA mutants. [file aem.01501-24-s0001.pdf]

Supplemental Information

Deciphering Reductive Dehalogenase Specificity Through Targeted Mutagenesis of Chloroalkane Reductases

Katherine Picott[a], Connor Bowers[a], and Elizabeth A. Edwards[a,b]#

[a]Department of Chemical Engineering and Applied Chemistry, University of Toronto, Toronto, Ontario, Canada

[b]Department of Cell and Systems Biology, University of Toronto, Toronto, Ontario, Canada

Running Head: RDase Specificity Changes from Key Mutations

#Address correspondence to Elizabeth A. Edwards, elizabeth.edwards@utoronto.ca.

SUPPLEMENTAL TEXT

Supplemental Text S1 – Ortholog Group 97 and Mutants ……………………………S4

Supplemental Text S2 – RDase Preparation ……………………………S8

Supplemental Text S3 – Activity Analysis ..…………………………S10

Supplemental Text S4 – Protein Models and Docking …………………..……S15

Supplemental Text S5 – DcrA Mutants …………………..……S20

SUPPLEMENTAL TABLES

Table S1 Primers for CfrA mutant construction ……………………………S8

Table S2 Additional expression plasmids ……………………………S9

Table S3 Docking results ………………………..…S16

Table S4 Steric strain of rotamers and mutants …………………………..S18

Table S5 Protein concentrations and purity Accompanying Excel

Table S6 Raw data for all main substrates presented Accompanying Excel

Table S7 Raw data for additional substrates tested Accompanying Excel

SUPPLEMENTAL FIGURES

Figure S1 SDS-PAGE of enzymes ……………………………S9

Figure S2 Dechlorination assays on CF, 1,1,1-TCA, and 1,1-DCA ……………………..….S11

Figure S3 Dechlorination assays on 1,1,2-TCA …………………………...S12

Figure S4 Dechlorination assays on DCM and 1,2-DCA …………………..……S13

Figure S5 Maximum-Likelihood tree and activity clustering …………………………...S14

Figure S6 CfrA mutant active site configuration …………………………...S17

Figure S7 CfrA, DcrA, AcdA, TmrA active site configuration …………………………...S17

Figure S8 CfrA mutant Tyr256 rotamer predictions …………………………...S18

Figure S9 CfrA, DcrA, AcdA, TmrA Tyr256 rotamer predictions …………………………...S19

Figure S10 $PceA_S$, $PceA_D$, CfrA, and DcrA arginine interactions …………………………...S19

Figure S11 $PceA_S$, $PceA_D$, CfrA, and DcrA residue 80 interactions …………………………...S19

Figure S12 DcrA mutant CF, 1,1,1-TCA, 1,1-DCA activity assays …………………………...S21

Figure S13 DcrA mutant 1,1,2-TCA activity assays …………………………...S22

Figure S14 DcrA mutants assay on DCM and 1,2-DCA ……….…………………S22

Figure S15 DcrA mutant variations around iron-sulfur clusters …………………………...S23

Figure S16 DcrA mutant active site configuration …………………………...S23

Figure S17 DcrA mutant Y256 rotamer predictions …………………………...S24

Supplemental Text S1 – Ortholog Group 97 and Mutants

Alignment of Ortholog Group 97 members and mutants used in this study. Green highlights show the positions which were mutated in the CfrA mutants and DcrA active site mutations. Blue highlights show the positions mutated in the DcrA mutants outside of the active site. Yellow highlights show any residues that are not in agreement with the consensus, the full column is highlighted if there is no consensus. Magenta highlighted residues are those predicted to interact with the cobamide cofactor in the AlphaFill models. All residues that interact with the cobamide are conserved except for residue 373 which is either a glutamine or histidine. The residues are numbered and the consensus sequence for just the Ortholog Group 97 members (mutants did not contribute to the consensus) is shown on the top.

> Amino acid alignment of
Dehalobacter sp. CF CfrA (AFV05253)
Dehalobacter sp. DCA DcrA (WP_015043247)
Dehalobacter sp. UNSWDHB TmrA (WP_034377773)
Dehalobacter sp. SAD AcdA (Accession not available) (1)
Dehalobacter sp. 8M RdhA D8M_v2_40029 (Accession not available) (2)
Dehalobacter sp. THM1 ThmA (ANI21407)
Desulfitobacterium sp. PR CtrA (AGO27983)

```
Consensus            MDKEKSNNDKPATKINRRRFLKFGAGASSGIAIAAAATALGGKSLIDPKQVYAGTVKELD
                     1........|.........|.........|.........|.........|.........60
CfrA                 MDKEKSNNDKPATKINRRQFLKFGAGASSGIAIATAATALGGKSLIDPKQVYAGTVKELD
DcrA                 MDKEKSNNDKPATKINRRQFLKFGAGASSGIAIATAATALGGKSLIDPKQVYAGTVKELD
CfrA-Y80W            MDKEKSNNDKPATKINRRQFLKFGAGASSGIAIATAATALGGKSLIDPKQVYAGTVKELD
CfrA-F125W           MDKEKSNNDKPATKINRRQFLKFGAGASSGIAIATAATALGGKSLIDPKQVYAGTVKELD
CfrA-Y256F           MDKEKSNNDKPATKINRRQFLKFGAGASSGIAIATAATALGGKSLIDPKQVYAGTVKELD
CfrA-C260Y           MDKEKSNNDKPATKINRRQFLKFGAGASSGIAIATAATALGGKSLIDPKQVYAGTVKELD
CfrA-M391W           MDKEKSNNDKPATKINRRQFLKFGAGASSGIAIATAATALGGKSLIDPKQVYAGTVKELD
CfrA-Y80W-F125W      MDKEKSNNDKPATKINRRQFLKFGAGASSGIAIATAATALGGKSLIDPKQVYAGTVKELD
CfrA-Y80W-Y256F      MDKEKSNNDKPATKINRRQFLKFGAGASSGIAIATAATALGGKSLIDPKQVYAGTVKELD
CfrA-Y80W-C260Y      MDKEKSNNDKPATKINRRQFLKFGAGASSGIAIATAATALGGKSLIDPKQVYAGTVKELD
CfrA-3M              MDKEKSNNDKPATKINRRQFLKFGAGASSGIAIATAATALGGKSLIDPKQVYAGTVKELD
CfrA-5M              MDKEKSNNDKPATKINRRQFLKFGAGASSGIAIATAATALGGKSLIDPKQVYAGTVKELD
DcrA-5M              MDKEKSNNDKPATKINRRQFLKFGAGASSGIAIATAATALGGKSLIDPKQVYAGTVKELD
DcrA-FeS             MDKEKSNNDKPATKINRRQFLKFGAGASSGIAIATAATALGGKSLIDPKQVYAGTVKELD
DcrA-AS-FeS          MDKEKSNNDKPATKINRRQFLKFGAGASSGIAIATAATALGGKSLIDPKQVYAGTVKELD
TmrA                 MDKEKSNNDKPATKINRRQFLKFGAGASSGIAIATAATALGGKSLIDPKQVYAGTVKELD
AcdA                 MDKEKSNNDKPATKINRRQFLKFGAGASSGIAIATAATALGGKSLIDPKQVYAGTVKELD
RdhA D8M_v2_40029    MDKEKSNNDKPATKINRRRFLKFGAGASSGIAIAAAATALGGKSLIDPKQVYAGTVKELD
ThmA                 MDKEKSNNDKPATKINRRRFLKFGAGASSGIAIAAAATALGGKSLIDPKQVYAGTVKELD
CtrA                 MDKEKSNNDKPATKINRRRFLKFGAGASSGIAIAAAATALGGKSLIDPKQVYAGTVKELD

Consensus            ELPFNIPADYKPFTNQRNIFGQAVLGVPEPLALVERFDEVRWNGWQTDGSPGLTVLDGAA
                   61........|.........|.........|.........|.........|.........120
CfrA                 ELPFNIPADYKPFTNQRNIYGQAVLGVPEPLALVERFDEVRWNGWQTDGSPGLTVLDGAA
DcrA                 ELPFNIPADYKPFTHQRNIWGQALLGVPEPLALRERFAEVRWNGWQTDGSPGLTVLDGAA
CfrA-Y80W            ELPFNIPADYKPFTNQRNIWGQAVLGVPEPLALVERFDEVRWNGWQTDGSPGLTVLDGAA
CfrA-F125W           ELPFNIPADYKPFTNQRNIYGQAVLGVPEPLALVERFDEVRWNGWQTDGSPGLTVLDGAA
CfrA-Y256F           ELPFNIPADYKPFTNQRNIYGQAVLGVPEPLALVERFDEVRWNGWQTDGSPGLTVLDGAA
CfrA-C260Y           ELPFNIPADYKPFTNQRNIYGQAVLGVPEPLALVERFDEVRWNGWQTDGSPGLTVLDGAA
```

```
CfrA-M391W            ELPFNIPADYKPFTNQRNIYGQAVLGVPEPLALVERFDEVRWNGWQTDGSPGLTVLDGAA
CfrA-Y80W-F125W       ELPFNIPADYKPFTNQRNIWGQAVLGVPEPLALVERFDEVRWNGWQTDGSPGLTVLDGAA
CfrA-Y80W-Y256F       ELPFNIPADYKPFTNQRNIWGQAVLGVPEPLALVERFDEVRWNGWQTDGSPGLTVLDGAA
CfrA-Y80W-C260Y       ELPFNIPADYKPFTNQRNIWGQAVLGVPEPLALVERFDEVRWNGWQTDGSPGLTVLDGAA
CfrA-3M               ELPFNIPADYKPFTNQRNIYGQAVLGVPEPLALVERFDEVRWNGWQTDGSPGLTVLDGAA
CfrA-5M               ELPFNIPADYKPFTNQRNIWGQAVLGVPEPLALVERFDEVRWNGWQTDGSPGLTVLDGAA
DcrA-5M               ELPFNIPADYKPFTHQRNIYGQALLGVPEPLALRERFAEVRWNGWQTDGSPGLTVLDGAA
DcrA-FeS              ELPFNIPADYKPFTHQRNIWGQALLGVPEPLALVERFAEVRWNGWQTDGSPGLTVLDGAA
DcrA-AS-FeS           ELPFNIPADYKPFTHQRNIYGQALLGVPEPLALRERFAEVRWNGWQTDGSPGLTVLDGAA
TmrA                  ELPFNIPADYKPFTNQRNIFGQAVLGVPEPLALVERFDEVRWNGWQTDGSPGLTVLDGAA
AcdA                  ELPFNIPADYKPFTNQRNIFGQAVLGVPEPLALQERFDEVRWNGWQTDGSPGLTVLDGAA
RdhA D8M_v2_40029     ELPFNIPADYKPFTNQRNIFGQAVLGVPEPLALVERFDEVRWNGWQTDGSPGLTVLDGAA
ThmA                  ELPFNIPADYKPFTNQRNIFGQALLGVPEPLALVERFDEVRWNGWQTDGSPGLTVLDGAA
CtrA                  ELPFNIPADYKPFTNQRNIFGQALLGVPEPRALVERFDEVRWNGWLTDGSPGLTVLDGAA

Consensus             ARASFAVDYYFNGENSACRANKGFFEWHPKVPELNFRWGDPERNIHSPGVKSAEEGTMAV

                    121........|.........|.........|.........|.........|.........180
CfrA                  ARASFAVDYYFNGENSACRANKGFFEWHPKVAELNFKWGDPERNIHSPGVKSAEEGTMAV
DcrA                  AHASWAVDYYLNGENSACRANKGFFEWHPKVPELNFRWGDPERNIHSPGVKSAEEGTMAV
CfrA-Y80W             ARASFAVDYYFNGENSACRANKGFFEWHPKVAELNFKWGDPERNIHSPGVKSAEEGTMAV
CfrA-F125W            ARASWAVDYYFNGENSACRANKGFFEWHPKVAELNFKWGDPERNIHSPGVKSAEEGTMAV
CfrA-Y256F            ARASFAVDYYFNGENSACRANKGFFEWHPKVAELNFKWGDPERNIHSPGVKSAEEGTMAV
CfrA-C260Y            ARASFAVDYYFNGENSACRANKGFFEWHPKVAELNFKWGDPERNIHSPGVKSAEEGTMAV
CfrA-M391W            ARASFAVDYYFNGENSACRANKGFFEWHPKVAELNFKWGDPERNIHSPGVKSAEEGTMAV
CfrA-Y80W-F125W       ARASWAVDYYFNGENSACRANKGFFEWHPKVAELNFKWGDPERNIHSPGVKSAEEGTMAV
CfrA-Y80W-Y256F       ARASFAVDYYFNGENSACRANKGFFEWHPKVAELNFKWGDPERNIHSPGVKSAEEGTMAV
CfrA-Y80W-C260Y       ARASFAVDYYFNGENSACRANKGFFEWHPKVAELNFKWGDPERNIHSPGVKSAEEGTMAV
CfrA-3M               ARASFAVDYYFNGENSACRANKGFFEWHPKVAELNFKWGDPERNIHSPGVKSAEEGTMAV
CfrA-5M               ARASWAVDYYFNGENSACRANKGFFEWHPKVAELNFKWGDPERNIHSPGVKSAEEGTMAV
DcrA-5M               AHASFAVDYYLNGENSACRANKGFFEWHPKVPELNFRWGDPERNIHSPGVKSAEEGTMAV
DcrA-FeS              AHASWAVDYYLNGENSACRANKGFFEWHPKVPELNFRWGDPERNIHSPGVKSAEEGTMAV
DcrA-AS-FeS           AHASFAVDYYLNGENSACRANKGFFEWHPKVPELNFRWGDPERNIHSPGVKSAEEGTMAV
TmrA                  ARASFAVDYYFNGENSACRANKGFFEWHPKVPELNFKWGDPERNIHSPGVKSAEEGTMAV
AcdA                  ARASFAVDYYLNGENSACRANKGFFEWHPKVPELNFKWGDPERNIHSPGVKSAEEGTMAV
RdhA D8M_v2_40029     ARASFAVDYYINGENSACRANKGFFEWHPKVPELNFKWGDPERNIHSPGVKSAEEGTMAV
ThmA                  ARASFAVDYYFNGENSACRANKGFFEWHPKVPELNFRWGDPERNIHSPGVKSAEEGTMAV
CtrA                  ARASFAVDYYFNGENSACRANKGFFEWHPKVPELNFRWGDPERNIHSPGVKSAEEGTMAV

Consensus             KXMARFFGAAKAGIAPFDKRWVFTETAAFVKTPEGESLKFIPPDFGFEPKHVISMIIPQS

                    181........|.........|.........|.........|.........|.........240
CfrA                  KKIARFFGAAKAGIAPFDKRWVFTETYAFVKTPEGESLKFIPPDFGFEPKHVISMIIPQS
DcrA                  KKIARFFGAAKAGIAPFDKRWVFTETAAFVKTPEGESLKFIPPDFGFEPKHVISMIIPQS
CfrA-Y80W             KKIARFFGAAKAGIAPFDKRWVFTETYAFVKTPEGESLKFIPPDFGFEPKHVISMIIPQS
CfrA-F125W            KKIARFFGAAKAGIAPFDKRWVFTETYAFVKTPEGESLKFIPPDFGFEPKHVISMIIPQS
CfrA-Y256F            KKIARFFGAAKAGIAPFDKRWVFTETYAFVKTPEGESLKFIPPDFGFEPKHVISMIIPQS
CfrA-C260Y            KKIARFFGAAKAGIAPFDKRWVFTETYAFVKTPEGESLKFIPPDFGFEPKHVISMIIPQS
CfrA-M391W            KKIARFFGAAKAGIAPFDKRWVFTETYAFVKTPEGESLKFIPPDFGFEPKHVISMIIPQS
CfrA-Y80W-F125W       KKIARFFGAAKAGIAPFDKRWVFTETYAFVKTPEGESLKFIPPDFGFEPKHVISMIIPQS
CfrA-Y80W-Y256F       KKIARFFGAAKAGIAPFDKRWVFTETYAFVKTPEGESLKFIPPDFGFEPKHVISMIIPQS
CfrA-Y80W-C260Y       KKIARFFGAAKAGIAPFDKRWVFTETYAFVKTPEGESLKFIPPDFGFEPKHVISMIIPQS
CfrA-3M               KKIARFFGAAKAGIAPFDKRWVFTETYAFVKTPEGESLKFIPPDFGFEPKHVISMIIPQS
CfrA-5M               KKIARFFGAAKAGIAPFDKRWVFTETYAFVKTPEGESLKFIPPDFGFEPKHVISMIIPQS
DcrA-5M               KKIARFFGAAKAGIAPFDKRWVFTETAAFVKTPEGESLKFIPPDFGFEPKHVISMIIPQS
DcrA-FeS              KKIARFFGAAKAGIAPFDKRWVFTETAAFVKTPEGESLKFIPPDFGFEPKHVISMIIPQS
DcrA-AS-FeS           KKIARFFGAAKAGIAPFDKRWVFTETAAFVKTPEGESLKFIPPDFGFEPKHVISMIIPQS
TmrA                  KRMARFFGAAKAGIAPFDKRWVFTETAAFVKTPEGEDLKFIPPDFGFEPKHVISMIIPQS
AcdA                  KKIARFFGAAKAGIAPFDKRWVFTETAAFVKTPEGESLKFIPPDFGFEPKHVISMIIPQS
RdhA D8M_v2_40029     KKMARFFGAAKAGIAPFDKRWVFTETAAFVKTPEGEDLKFIPPDFGFEPKHVISMIIPQS
ThmA                  KRMARFFGAAKAGIAPFDKRWVFTETAAFVKTPEGESLKFIPPDFGFEPKHVISMIIPQS
CtrA                  KRMARFFGAAKAGIAPFDKRWVFTETAAFVKTPEGESLKFIPPDFGFEPKHVISMIIPQS
```

```
Consensus            LEGVKCAPSFLGSAEYGLSCAQIGYAAFGLSMFIKDLGYHAVPIGADSALAIPIAIQAGL

                    241........|.........|.........|.........|.........|.........300
CfrA                 PEGVKCDPSFLGSTEYGLSCAQIGYAAFGLSMFIKDLGYHAVPIGSDSALAIPIAIQAGL
DcrA                 LEGTKCAPSFLGSAEYGLSYTQIGYAAFGLSMFIKDLGYHAVPIGADSALAIPIAIQAGL
CfrA-Y80W            PEGVKCDPSFLGSTEYGLSCAQIGYAAFGLSMFIKDLGYHAVPIGSDSALAIPIAIQAGL
CfrA-F125W           PEGVKCDPSFLGSTEYGLSCAQIGYAAFGLSMFIKDLGYHAVPIGSDSALAIPIAIQAGL
CfrA-Y256F           PEGVKCDPSFLGSTEFGLSCAQIGYAAFGLSMFIKDLGYHAVPIGSDSALAIPIAIQAGL
CfrA-C260Y           PEGVKCDPSFLGSTEYGLSYAQIGYAAFGLSMFIKDLGYHAVPIGSDSALAIPIAIQAGL
CfrA-M391W           PEGVKCDPSFLGSTEYGLSCAQIGYAAFGLSMFIKDLGYHAVPIGSDSALAIPIAIQAGL
CfrA-Y80W-F125W      PEGVKCDPSFLGSTEYGLSCAQIGYAAFGLSMFIKDLGYHAVPIGSDSALAIPIAIQAGL
CfrA-Y80W-Y256F      PEGVKCDPSFLGSTEFGLSCAQIGYAAFGLSMFIKDLGYHAVPIGSDSALAIPIAIQAGL
CfrA-Y80W-C260Y      PEGVKCDPSFLGSTEYGLSYAQIGYAAFGLSMFIKDLGYHAVPIGSDSALAIPIAIQAGL
CfrA-3M              PEGVKCDPSFLGSTEYGLSYAQIGYAAFGLSMFIKDLGYHAVPIGSDSALAIPIAIQAGL
CfrA-5M              PEGVKCDPSFLGSTEYGLSYAQIGYAAFGLSMFIKDLGYHAVPIGSDSALAIPIAIQAGL
DcrA-5M              LEGTKCAPSFLGSAEYGLSCTQIGYAAFGLSMFIKDLGYHAVPIGADSALAIPIAIQAGL
DcrA-FeS             LEGVKCDPSFLGSAEYGLSYTQIGYAAFGLSMFIKDLGYHAVPIGADSALAIPIAIQAGL
DcrA-AS-FeS          LEGVKCAPSFLGSAEYGLSYTQIGYAAFGLSMFIKDLGYHAVPIGADSALAIPIAIQAGL
TmrA                 LEGVKCAPSFLGSAEYGLSFAQIGYAAFGLSMFIKDLGYHAVPIGSDSALSIPIAIQAGL
AcdA                 LEGIKCAPSFLGSAEYGLSYAQIGYAAFGLSMFIKDLGYHAVPIGADSALAVPIAIQAGL
RdhA D8M_v2_40029    LEGVKCAPSFLGSAEYGLSFAQIGYAAFGLSMFIKDLGYHAVPTGSDSALAIPIAIQAGL
ThmA                 LEGVKSAPSFLGSSEYGLSCAQYGYAPFGLSMFIKDLGYHAVPIGADSALAIPIAIQAGL
CtrA                 LEGVKTSPSFLGSSEYGLGCAQYGYAPFGLSMFIKDLGYHAVPIGADSALAIPIAIQAGL

Consensus            GEYSRSGLMITPEFGPNVRLCEVFTDMPLNHDKPISFGVTEFCKTCKKCAEACAPQAISY

                    301........|.........|.........|.........|.........|.........360
CfrA                 GEYSRSGLMITPEFGSNVRLCEVFTDMPLNHDKPISFGVTEFCKTCKKCAEACAPQAISY
DcrA                 GEYSRSGLMITPEFGPNVRLCEVFTDMPLNHDKPISFGVTEFCKTCKKCAEACAPQAISY
CfrA-Y80W            GEYSRSGLMITPEFGSNVRLCEVFTDMPLNHDKPISFGVTEFCKTCKKCAEACAPQAISY
CfrA-F125W           GEYSRSGLMITPEFGSNVRLCEVFTDMPLNHDKPISFGVTEFCKTCKKCAEACAPQAISY
CfrA-Y256F           GEYSRSGLMITPEFGSNVRLCEVFTDMPLNHDKPISFGVTEFCKTCKKCAEACAPQAISY
CfrA-C260Y           GEYSRSGLMITPEFGSNVRLCEVFTDMPLNHDKPISFGVTEFCKTCKKCAEACAPQAISY
CfrA-M391W           GEYSRSGLMITPEFGSNVRLCEVFTDMPLNHDKPISFGVTEFCKTCKKCAEACAPQAISY
CfrA-Y80W-F125W      GEYSRSGLMITPEFGSNVRLCEVFTDMPLNHDKPISFGVTEFCKTCKKCAEACAPQAISY
CfrA-Y80W-Y256F      GEYSRSGLMITPEFGSNVRLCEVFTDMPLNHDKPISFGVTEFCKTCKKCAEACAPQAISY
CfrA-Y80W-C260Y      GEYSRSGLMITPEFGSNVRLCEVFTDMPLNHDKPISFGVTEFCKTCKKCAEACAPQAISY
CfrA-3M              GEYSRSGLMITPEFGSNVRLCEVFTDMPLNHDKPISFGVTEFCKTCKKCAEACAPQAISY
CfrA-5M              GEYSRSGLMITPEFGSNVRLCEVFTDMPLNHDKPISFGVTEFCKTCKKCAEACAPQAISY
DcrA-5M              GEYSRSGLMITPEFGPNVRLCEVFTDMPLNHDKPISFGVTEFCKTCKKCAEACAPQAISY
DcrA-FeS             GEYSRSGLMITPEFGSNVRLCEVFTDMPLNHDKPISFGVTEFCKTCKKCAEACAPQAISY
DcrA-AS-FeS          GEYSRSGLMITPEFGPNVRLCEVFTDMPLNHDKPISFGVTEFCKTCKKCAEACAPQAISY
TmrA                 GEYSRSGQMITPEFGPNVRLCEVFTDMPLNHDKPISFGVTEFCKTCKKCAEACPPQAISY
AcdA                 GEYSRSGLMITPEFGSNVRLCEVFTDMPLNHDKPISFGVTEFCKTCKKCAEACPPQAISY
RdhA D8M_v2_40029    GEYSRSGQMITPEFGSNVRLCEVFTDMPLNHDKPISFGVTEFCKTCKKCAEACPPQAISY
ThmA                 GEYSRLGLMITPEFGPNVRLCEVFTDMPLNHDKPISFGVTEFCKTCKKCAEACAPQAISY
CtrA                 GEYSRMGLMITPEFGPNVRLCEVFTDMPLNHDKPISFGVTEFCKTCKKCAEECAPQAISY

Consensus            EDPTIDGPRGQMQNSGIKRWYVDPVKCXEFWSRDNVRNCCGACIAACPFTKPEAWHHTLI

                    361........|.........|.........|.........|.........|.........420
CfrA                 EDPTIDGPRGQMQNSGIKRWYVDPVKCLEFMSRDNVGNCCGACIAACPFTKPEAWHHTLI
DcrA                 EDPTIDGPRGQMQNSGIKRWYVDPVKCFEFWSRDNVRNCCGACIAACPFTKPEAWHHTLI
CfrA-Y80W            EDPTIDGPRGQMQNSGIKRWYVDPVKCLEFMSRDNVGNCCGACIAACPFTKPEAWHHTLI
CfrA-F125W           EDPTIDGPRGQMQNSGIKRWYVDPVKCLEFMSRDNVGNCCGACIAACPFTKPEAWHHTLI
CfrA-Y256F           EDPTIDGPRGQMQNSGIKRWYVDPVKCLEFMSRDNVGNCCGACIAACPFTKPEAWHHTLI
CfrA-C260Y           EDPTIDGPRGQMQNSGIKRWYVDPVKCLEFMSRDNVGNCCGACIAACPFTKPEAWHHTLI
CfrA-M391W           EDPTIDGPRGQMQNSGIKRWYVDPVKCLEFWSRDNVGNCCGACIAACPFTKPEAWHHTLI
CfrA-Y80W-F125W      EDPTIDGPRGQMQNSGIKRWYVDPVKCLEFMSRDNVGNCCGACIAACPFTKPEAWHHTLI
CfrA-Y80W-Y256F      EDPTIDGPRGQMQNSGIKRWYVDPVKCLEFMSRDNVGNCCGACIAACPFTKPEAWHHTLI
CfrA-Y80W-C260Y      EDPTIDGPRGQMQNSGIKRWYVDPVKCLEFMSRDNVGNCCGACIAACPFTKPEAWHHTLI
```

```
CfrA-3M               EDPTIDGPRGQMQNSGIKRWYVDPVKCFEFWSRDNVGNCCGACIAACPFTKPEAWHHTLI
CfrA-5M               EDPTIDGPRGQMQNSGIKRWYVDPVKCFEFWSRDNVGNCCGACIAACPFTKPEAWHHTLI
DcrA-5M               EDPTIDGPRGQMQNSGIKRWYVDPVKCLEFMSRDNVRNCCGACIAACPFTKPEAWHHTLI
DcrA-FeS              EDPTIDGPRGQMQNSGIKRWYVDPVKCFEFWSRDNVRNCCGACIAACPFTKPEAWHHTLI
DcrA-AS-FeS           EDPTIDGPRGQMQNSGIKRWYVDPVKCFEFWSRDNVRNCCGACIAACPFTKPEAWHHTLI
TmrA                  EDPTIDGPRGQMHNSGIKRWYVDPVKCFEFWSRDNVRNCCGACIAACPFTKPEAWHHTLI
AcdA                  EDPTIDGPRGQMHNSGIKRWYVDPVKCFEFWSRDNVRNCCGACIAACPFTKPEAWHHTLI
RdhA D8M_v2_40029     EDPTIDGPCGQMHNSGIKRWYVDPVKCFEFWSRDNVRNCCGACIAACPFTKPEAWHHTLT
ThmA                  EDPTIDGPRGQMQNSGIKRWYVDPVKCLEFWSRDNVRNCCGACIAACPFTKPEAWHHTLI
CtrA                  EDPTIDGPRGQMQNSGIKRWYVDPVKCLEFWSRDNVRDCCGACIAACPFTKPEAWHHTLI

Consensus             RSLVGAPVITPFMKDMDDIFGY-GKPNDEKAIADWWK

                    421........|.........|.........|......457
CfrA                  RSLVGAPVITPFMKDMDDIFGY-GKLNDEKAIADWWK
DcrA                  RSLVGAPVITPFMKDMDDIFGY-GKPNDEKAIADWWK
CfrA-Y80W             RSLVGAPVITPFMKDMDDIFGY-GKLNDEKAIADWWK
CfrA-F125W            RSLVGAPVITPFMKDMDDIFGY-GKLNDEKAIADWWK
CfrA-Y256F            RSLVGAPVITPFMKDMDDIFGY-GKLNDEKAIADWWK
CfrA-C260Y            RSLVGAPVITPFMKDMDDIFGY-GKLNDEKAIADWWK
CfrA-M391W            RSLVGAPVITPFMKDMDDIFGY-GKLNDEKAIADWWK
CfrA-Y80W-F125W       RSLVGAPVITPFMKDMDDIFGY-GKLNDEKAIADWWK
CfrA-Y80W-Y256F       RSLVGAPVITPFMKDMDDIFGY-GKLNDEKAIADWWK
CfrA-Y80W-C260Y       RSLVGAPVITPFMKDMDDIFGY-GKLNDEKAIADWWK
CfrA-3M               RSLVGAPVITPFMKDMDDIFGY-GKLNDEKAIADWWK
CfrA-5M               RSLVGAPVITPFMKDMDDIFGY-GKLNDEKAIADWWK
DcrA-5M               RSLVGAPVITPFMKDMDDIFGY-GKPNDEKAIADWWK
DcrA-FeS              RSLVGAPVITPFMKDMDDIFGY-GKPNDEKAIADWWK
DcrA-AS-FeS           RSLVGAPVITPFMKDMDDIFGY-GKPNDEKAIADWWK
TmrA                  RSLVGAPVITPFMKDMDDIFGY-GKPN-EKAKADWWK
AcdA                  RSLVGAPVITPFMKDVDDIFGY-GKPNDEKAIADWWK
RdhA D8M_v2_40029     RSLAGAPVITPFMKNLDDIFGY-GKPNDEKAIADWWK
ThmA                  RSLVGAPVITPFMKDMDDIFGY-GKPNDEKAIADWWK
CtrA                  RSLVGAPVITPFMKDMDDIFGYGGKPNDKKAIADWWK
```

Supplemental Text S2 – RDase Preparation

This section contains the details of the primers used to construct CfrA point mutant plasmids (Table S1), any expression plasmids used that were not included in the main text (Table S2), and the SDS-PAGE of each RDase tested in activity assays (Figure S1). The construction of the TmrA and AcdA expression plasmids are described elsewhere (1, 3). The *pBAD42-BtuCEDFB* plasmid was generously provided by the Booker Lab (Pennsylvania State University, PA, USA). The gene for DcrA-FeS and the gene for DcrA-AS-FeS in *pET-21* were purchased from Twist Bioscience (San Francisco, CA, USA). The DcrA-FeS gene was cloned into *p15TV-L* as described in the main text.

The SDS-PAGE was used to estimate the purity of the RDase in the preparation. The yellow block indicates where the RDase band is located (top band in box). The lower bands are predicted to be degraded forms of the RDase, but this is unconfirmed. Only the top band in each case was used for the purity estimate. All of the concentrations were measured using a Bradford assay and adjusted with the purity, all concentration and purity details can be found in Table S5 in the accompanying Excel document.

Table S1. Primers used for site-directed mutagenesis of CfrA. Red indicates the mutated codon.

Primer Name	Sequence (3' → 5')	Resulting Mutation
CfrA_Y80W_F	CCAATCAAAGGAATATA**TGG**GGCCAGGCTGTATTGGGAG	Y80W
CfrA_Y80W_R	CTCCCAATACAGCCTGGCC**CCA**TATATTCCTTTGATTGG	
CfrA_F125W_F	GCTGCTCGTGCAAGC**TGG**GCCGTTGATTATTATTTTAAC	F125W
CfrA_F125W_R	GTTAAAATAATAATCAACGGC**CCA**GCTTGCACGAGCAGC	
CfrA_C260Y_F	GGATTAAGT**TAT**GCCCAGATTGGATATGCTGC	C260Y
CfrA_C26Y_R	GCAGCATATCCAATCTGGGC**ATA**ACTTAATCC	
CfrA_Y256F_F	GGATCAACTGAA**TTT**GGATTAAGTTGTGCCCAG	Y256F
CfrA_Y256F_R	CTGGGCACAACTTAATCC**AAA**TTCAGTTGATCC	

Table S2. Enzymes that were expressed but not included in the main text.

Enzyme Expressed	Plasmid	Description
BtuCEDFB	*pBAD42-BtuCEDFB* [a]	Btu transport pathway under arabinose control.
TmrA	*p15TVL-tmrA*	Wild-type TmrA
AcdA	*p15TVL-acdA*	Wild-type AcdA
DcrA-FeS	*p15TVL-dcrA-FeS* [b]	DcrA with mutations: R94V, T244V, A247D, and P316S, all located near [4Fe-4S] clusters
DcrA-AS-FeS	*pET21-dcrA-AS-FeS* [c]	DcrA with mutations: T244V, W80Y, W125F

[a] provided by Booker lab(4),
[b] codon-optimized gene from Twist Bioscience then assembled into *p15TV-L*
[c] codon-optimized and cloned into *pET- 21* from Twist Bioscience

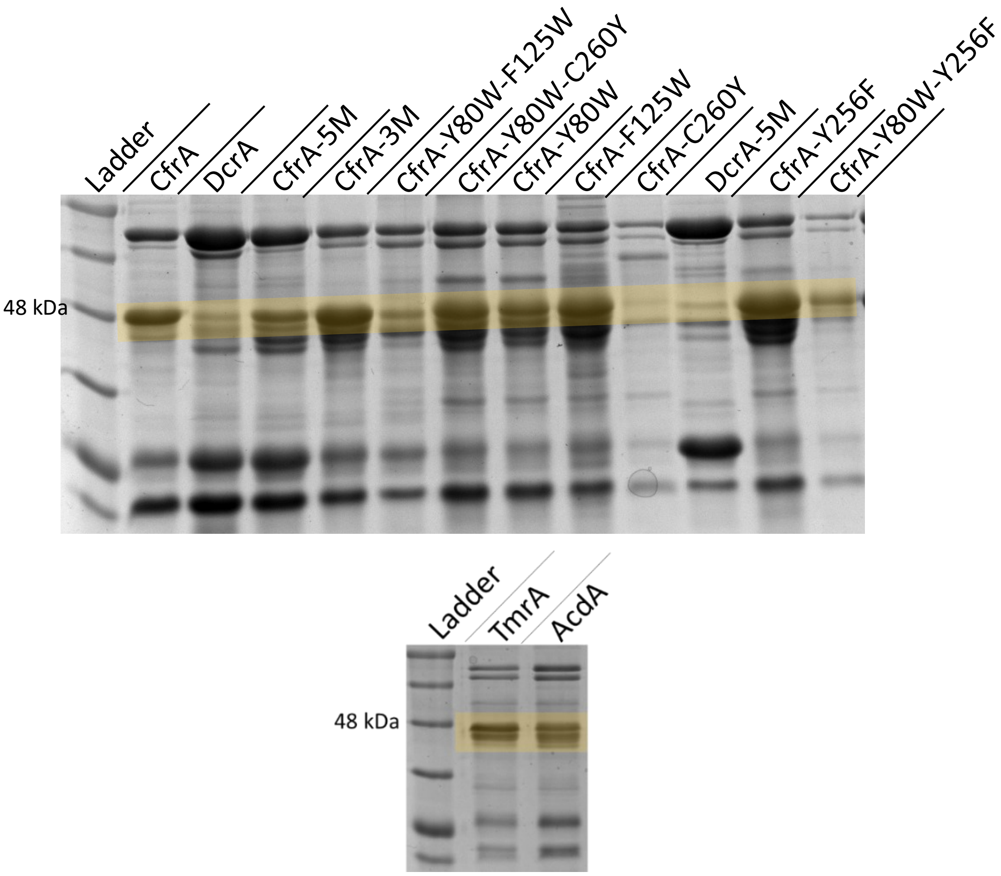

Figure S1. SDS-PAGE of all proteins used in enzyme assays. The samples loaded are the concentrated proteins post-nickel affinity chromatography. The ladder used was FroggaBio BluEye pre-stained protein ladder. The yellow box indicates where the expected RDase band is (all are ~48 kDa).

Supplemental Text S3 – Additional Activity Analysis

Table S6, provided in the accompanying Excel document, contains all the raw data for the assays and negative controls. None of the enzyme-free negative controls had detectable products with the exception of one CF and one 1,1-DCA sample, which each had less than 0.15 nmol DCM and VC, respectively. Abiotic controls with reduced cobalamin did not result in dechlorination of 1,1,1-TCA or 1,1-DCA. The reduced cobalamin did produce 0.001 ± 0.0005 min^{-1} DCM from CF, much less than the RDases which ranged 2-200 min^{-1}. The cobalamin also transformed 1,1,2-TCA to 0.60 ± 0.03 min^{-1} VC, while the RDases have activity ranging from 3-180 min^{-1}. The full activity including all CfrA mutants, the negative controls, and TmrA and AcdA are shown in Figure S2 and Figure S3, and the rate values are in Table S6.

The bubble plot in the main text Figure 2D and Figure S2D were created by normalizing the activity of each enzyme to its highest activity using Equation 1. Where $S_{activity}$ is the specific activity of a particular enzyme on any given substrate and $S_{max\ activity}$ is the maximum activity measured by that enzyme. As such, the substrate that the enzyme is the most active on will have a normalized activity of 1.

Equation 1

$$S_{normalized\ activity} = \frac{S_{activity}}{S_{max\ activity}}$$

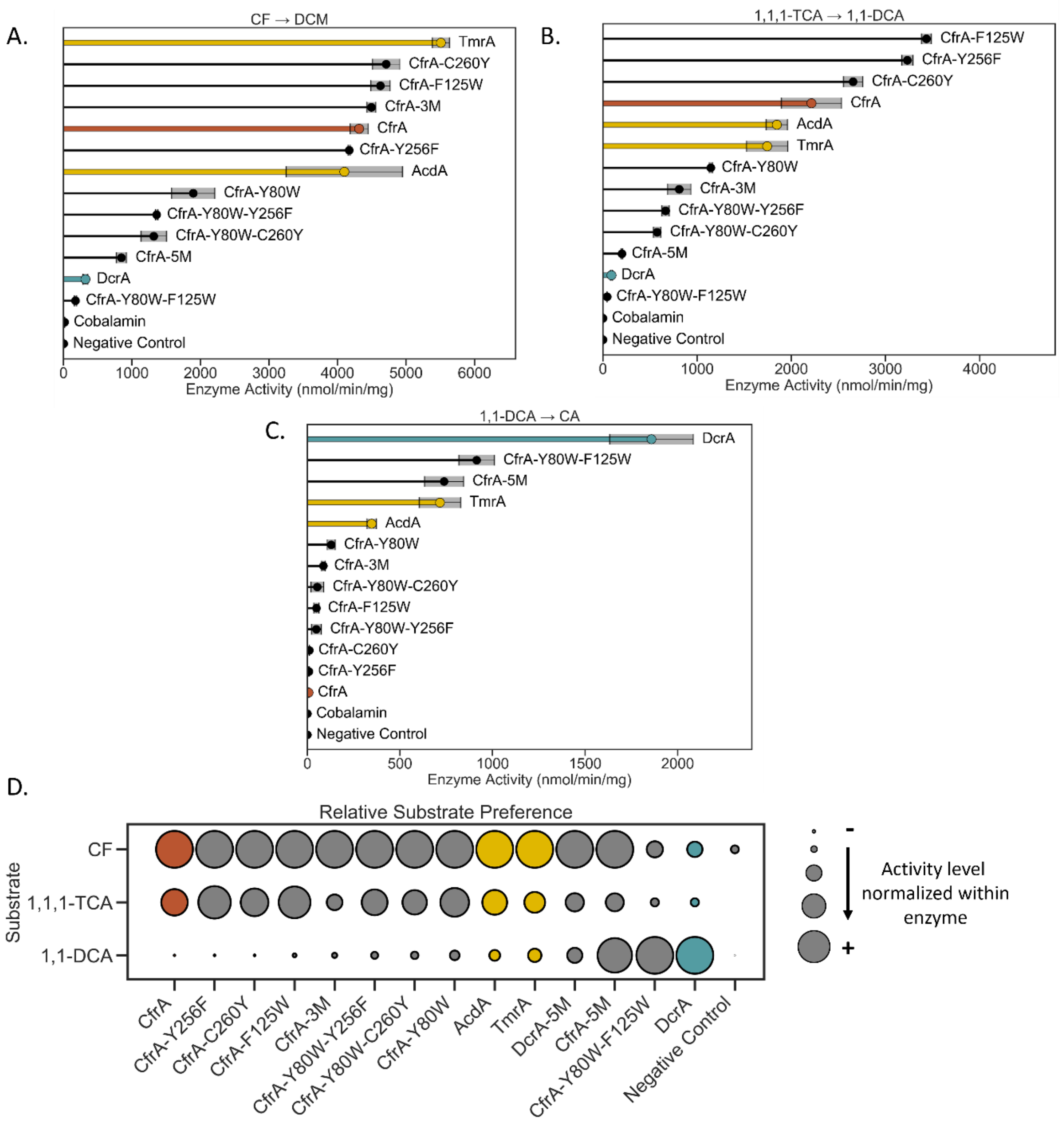

Figure S2. Dechlorination activity on (A) chloroform, (B) 1,1,1-trichloroethane, and (C) 1,1-dichloroethane after 1 hr. CfrA activity is highlighted in orange, DcrA is highlighted in blue, and TmrA and AcdA in yellow (D) The activity of each enzyme scaled from 0 to the substrate with the highest activity to show the relative substrate preferences, larger circles indicate higher activity levels. Grey shading indicates the standard deviation between samples (n = 3, n = 4 for CfrA and DcrA). CF = chloroform, DCM = dichloromethane, TCA = trichloroethane, DCA = dichloroethane, CA = chloroethane.

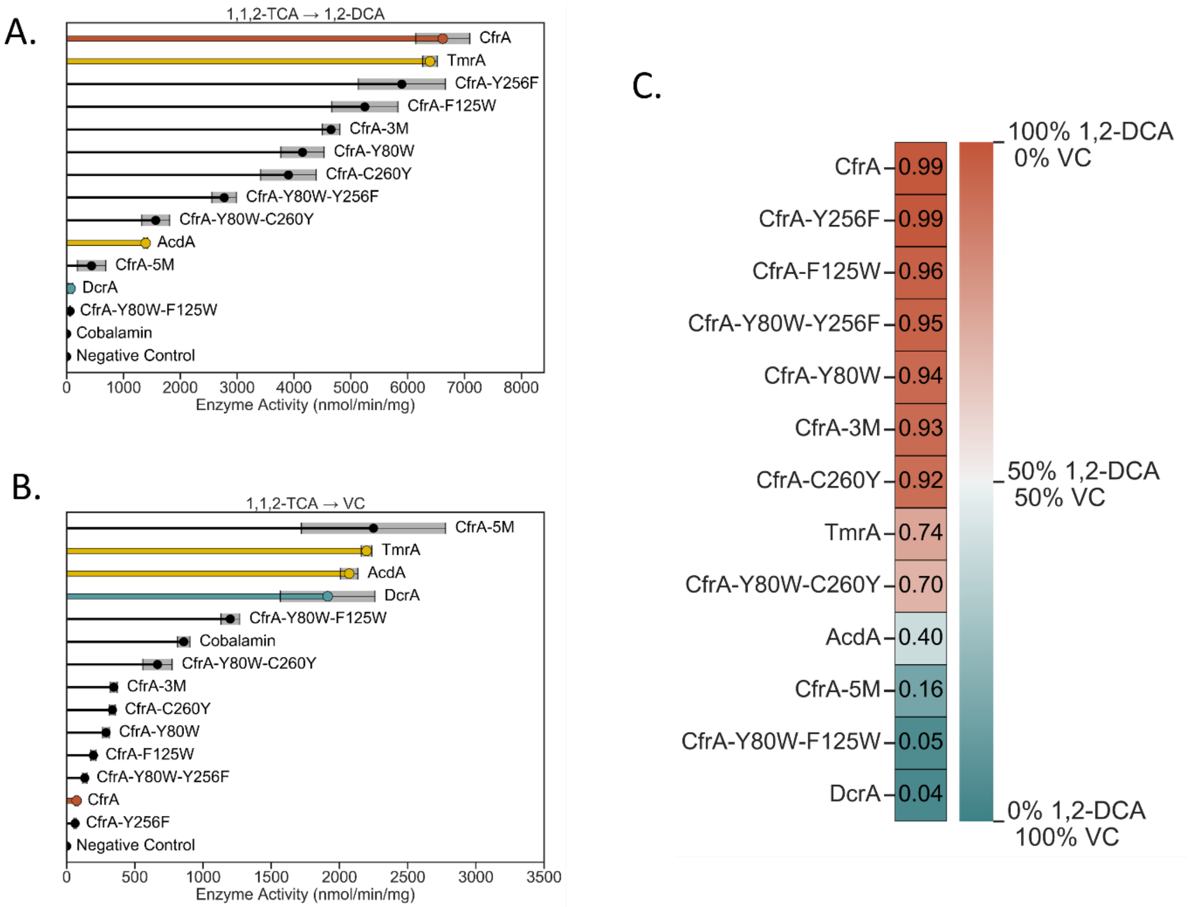

Figure S3. Dechlorination activity on 1,1,2-trichloroethane to produce (A) 1,2-dichloroethane or (B) vinyl chloride after 1 hr. (C) The proportion of the dechlorination product that went to 1,2-dichloroethane (more orange) and vinyl chloride (more blue); the number in the square indicates the ratio of 1,2-dichloroethane product. CfrA activity is highlighted in orange, DcrA in blue, and TmrA and AcdA in yellow. Grey shading indicates the standard deviation between samples (n = 3, n = 4 for CfrA and DcrA). TCA = trichloroethane, DCA = dichloroethane, VC = vinyl chloride.

Table S7, in the accompanying Excel document, has raw results for additional assays done using DCM and 1,2-DCA as substrates. Not all CfrA mutants were tested on these substrates. In general, DcrA has the highest level of activity on these substrates but (Figure S4), it still has a 10-fold higher activity on its native substrate, 1,1-DCA. CfrA-Y80W-F125W has the most increase from wild-type activity compared to CfrA-5M and CfrA-3M; again, this is still a very modest activity.

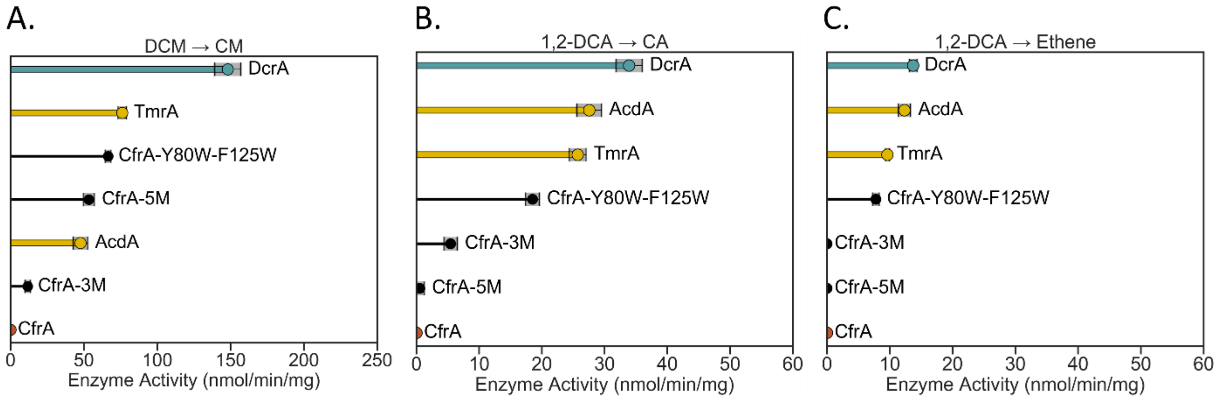

Figure S4. Dechlorination assay of dichloromethane (DCM; A) to chloromethane (CM) and 1,2-dichloroethane (DCA) to chloroethane (CA; B) and ethene (C) after 1 hr. CfrA is highlighted in red and DcrA in blue. Error bars are standard deviation between triplicate reactions. Note that the X-axis scale is different from previous figures as activities are very low.

Maximum-Likelihood Tree and Activity Clustering

The difference between the sequence and activity similarity of the CfrA mutants and the wild-type OF 97 RDases was visualized using a maximum-likelihood tree and a clustering dendrogram.

To cluster the enzymes based on relative activity on each substrate rather than absolute activity, the activities were normalized to the activity of CfrA and DcrA depending on the substrate. For reactions that are preferred by CfrA (CF, 1,1,1-TCA, 1,1,2-TCA→1,2-DCA), the activity was scaled such that CfrA had an activity of 1 and DcrA had an activity of 0 (Equation 2), and vice versa for reactions preferred by DcrA (1,1-DCA, 1,1,2-TCA→VC; Equation 3). The rest of the enzymes were normalized to the scale of CfrA and DcrA activity. In Equation 2 and Equation 3, $x_{activity}$ is the raw activity of enzyme x and $x_{normalized}$ is the activity normalized on the scale of CfrA–DcrA activity. Activities on DCM and 1,2-DCA were not included in the dendrogram construction.

Equation 2
$$x_{normalized} = \frac{x_{activity} - DcrA_{activity}}{CfrA_{activity} - DcrA_{activity}}$$

Equation 3
$$x_{normalized} = \frac{x_{activity} - CfrA_{activity}}{DcrA_{activity} - CfrA_{activity}}$$

The enzyme activity dendrogram was produced with the SciPy cluster package using the hierarchy function (5). A linkage matrix was constructed using the ‘average’ method for distance measuring and Euclidean distance metric.

A maximum-likelihood tree of the OG 97 enzymes and the mutants of interest was constructed in Geneious v8.1.9. First, a multiple sequence alignment of the amino acid sequences was created using the MAFFT v7.017 plugin using the BLOSUM80 scoring matrix and default penalty settings. The alignment

was used to produce a maximum-likelihood tree with the RAxML v7.2.8 plugin using the Gamma GTR model and 100 bootstraps; the consensus tree was used. The resulting tree was formatted with FigTree v1.4.4 and Adobe Illustrator 2024.

The CfrA mutants cluster in three groups based on their normalized activity (Figure S5), one group clustered with wild-type CfrA (red), one with DcrA (blue), and the other is not clustered with any natural RDases (yellow). The group that clusters with DcrA include CfrA-5M and CfrA-Y80W-F125W demonstrating the importance of both residue 80 and 125 in the activity determination. To support this, the majority of the yellow cluster has the Y80W mutation, with the exception of CfrA-3M. On their own, mutations at residues 125, 256, and 260 do not have much influence on CfrA activity as they still cluster with the wild-type enzyme.

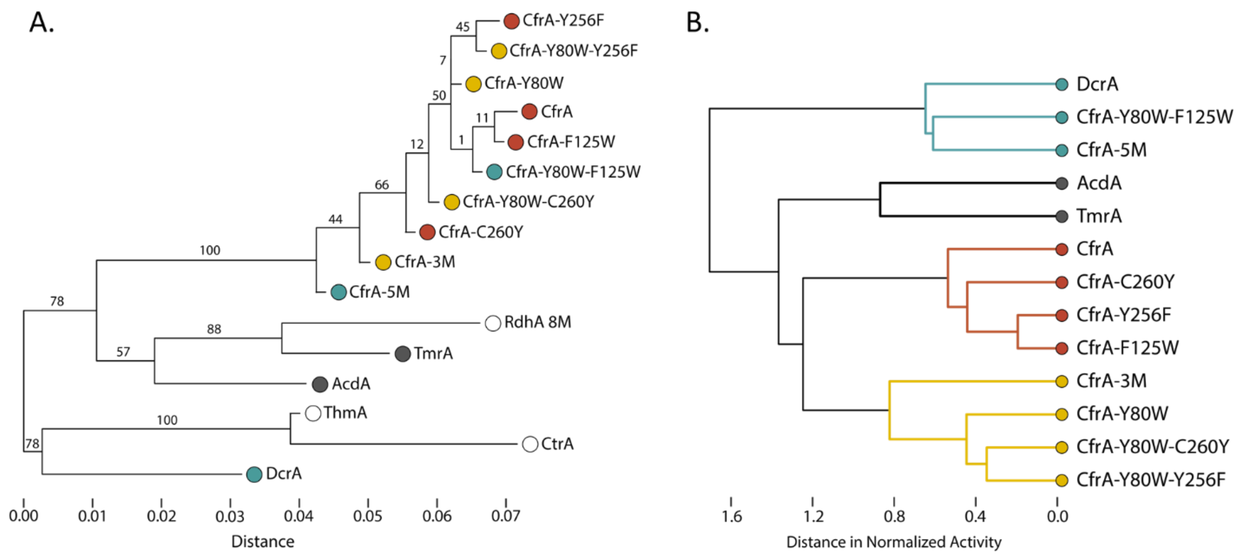

Figure S5. (A) Maximum-likelihood (ML) tree of OG 97 RDases and CfrA mutants and (B) CfrA mutant clustering by normalized activity. The coloured circles in the ML trees indicate the activity cluster that the enzyme belongs to in (B). Empty circles indicate sequences where the enzyme activity was not tested. The branch numbers are the number of bootstrap trees that agree with the branch split. The separate colour of activity clusters was determined with a cutoff of 1 distance unit in the normalized activity.

Supplemental Text S4 – Protein Models and Docking

The protein structure models of CfrA, DcrA, TmrA, AcdA, and the first round of CfrA mutants (CfrA-5M, CfrA-3M, CfrA-Y80W-F125W, CfrA-Y80W, and CfrA-F125W) were all docked with the four substrates tested in the activity assays (CF, 1,1,1-TCA, 1,1,2-TCA, and 1,1-DCA). The methods for docking were described in the main text, this section shows all the details of each docking with the residues found to interact with the docked substrate and the binding energy in Table S3. Notably, all of the CfrA dockings (and its mutants) had interactions between Tyr256 and the substrate, and this is not present in DcrA, TmrA, or AcdA. The residue interactions in the mutants are less reliable because these were constructed by superimposing the cobalamin in the structure, so the rotamer may not have taken account of the space taken up by the cobalamin in the active site. DcrA has a consistent interaction with Trp80 and all of the ligands, which correlates well with the idea that residue 80 gives additional bulk to the active site and stabilizes smaller residues. This interaction is not observed in the CfrA mutants with Y80W however.

The changes in steric strain with different rotamers (for the target residues) were assessed in the mutants against the wild-type CfrA and DcrA models using the PyMOL Wizard Mutagenesis plugin tool (Table S4). This tool allows for changes of individual residues to a different side-chain or a change in the residue rotamer. Further, the tool will give information on the strain of each rotamer and clashes with neighbouring residues (this will give higher strain values). The steric strain of each predicted rotamer in CfrA and DcrA was assessed with the mutagenesis tool (with no mutation introduced); higher values correspond to higher degrees of strain and less favourable conformations. The CfrA mutants were constructed based on the CfrA model with specific changes introduced with the mutagenesis tool. The rotamer of each mutated residue was selected as the one with the lowest steric strain and highest likelihood of observing the rotamer (likelihood is based on the population of the rotamers seen in crystal structures). The rotamers of the non-mutated residues were also changed to be the lowest strain rotamer. Finally, the two observed rotamers of Tyr256 were also assessed to compare the relative strain in the wild-type structures.

Generally, the introduction of mutations into the CfrA structure increases the strain observed in the mutated and neighbouring residues. The F125W and C260Y mutations increase the strain of the Tyr256 rotamer that points into the active site and thus may force Tyr256 out of the active site. This strain can also be seen by the clashing in the main text, Figure 5. The Y80W mutation does not have a large impact on the strain observed in any of the residues, which may be due to the side chain bulk going into the active site and decreasing the space for the substrate.

Visualization of the CfrA mutants CfrA-5M, CfrA-3M, and CfrA-Y80W-F125W, and the wild-type OG 97 RDase target active site residues can be seen in Figure S6 and Figure S7. The predicted configuration of Tyr256 in the AlphaFold structures of the mutants and wild-type RDases can be seen in Figure S8 and Figure S9. CfrA is the only wild-type RDase shown where Tyr256 is predicted to be oriented toward the active site and introducing the C260Y mutation causes Tyr256 to flip back in the predictive models.

Table S3. Results for CfrA, DcrA, TmrA, AcdA, and select mutants docked with CF, 1,1,1-TCA, 1,1-DCA, and 1,1,2-TCA. The table shows the residues that interact with the substrate and the binding energy.

	**CF**		**1,1,1-TCA**		**1,1-DCA**		**1,1,2-TCA**	
	**Residues**	**Energy kcal/mol**	**Residues**	**Energy kcal/mol**	**Residues**	**Energy kcal/mol**	**Residues**	**Energy kcal/mol**
CfrA-5M	Y260, Y256, R319	-3.64	S136, Y260, Y256, R319	-3.21	Y260, Y256, R319	-3.76	S136, Y256, Y260, S286	-3.91
CfrA-3M	Y260, Y256, R319	-3.93	S136, Y260, Y256, R319	-2.9	Y260, Y256, R319	-3.86	S136, Y256, Y260	-3.99
CfrA-Y80W-F125W	Y256	-3.50	S136, Y256	-3.87	Y256, S286	-3.45	S136, Y256, R319	-3.66
CfrA-Y80W	Y256, S286	-3.19	S136, Y256, C260, I263, S286	-2.84	Y256, S286	-3.15	Y256, C260, R319	-3.36
CfrA-F125W	S136, Y256, S286	-3.53	S136, Y256, R319	-3.90	S136, Y256, S286, R319	-3.48	S136, Y256, R319	-3.81
CfrA	S136, Y256, S286, R319	-3.38	S136, Y256, S286	-2.87	S136, Y256, S286, R319	-3.40	S136, Y256, S286, R319	-3.51
DcrA	W80	-3.47	W80, S136, W391	-3.84	W80, S136	-3.41	W80, A286, R319, W391	-3.69
TmrA	F80, W391	-3.63	F80, F260, W391	-3.63	W391	-3.57	F80, W391, R319	-3.95
AcdA	F80, S136, Y260, W391	-3.16	F80, S136, Y260, W391	-3.16	F80, S136, C138 Y260, W391	-3.30	F80, S136, Y260, W391	-3.67

CF = chloroform, TCA = trichloroethane, DCA = dichloroethane

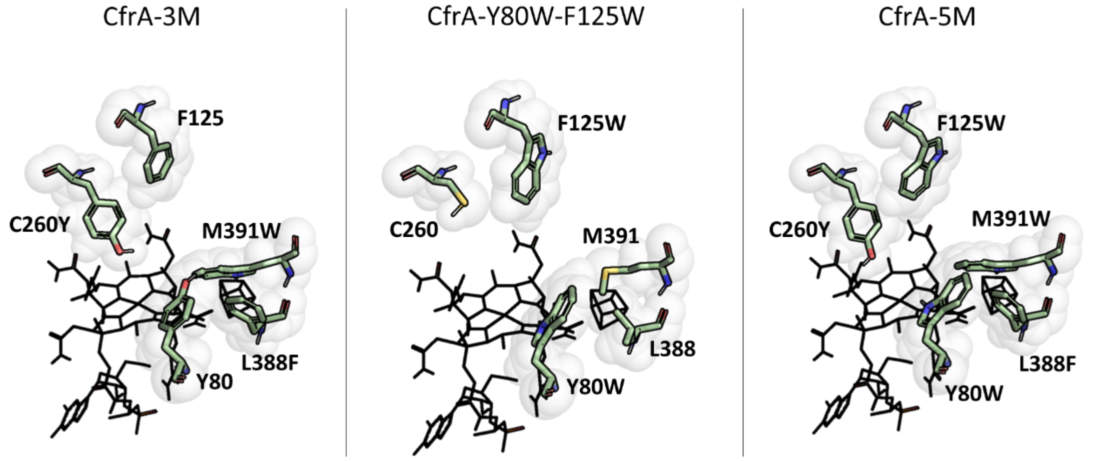

Figure S6. Active site configuration of CfrA-3M (left), CfrA-Y80W-F125W (center), and CfrA-5M (right).

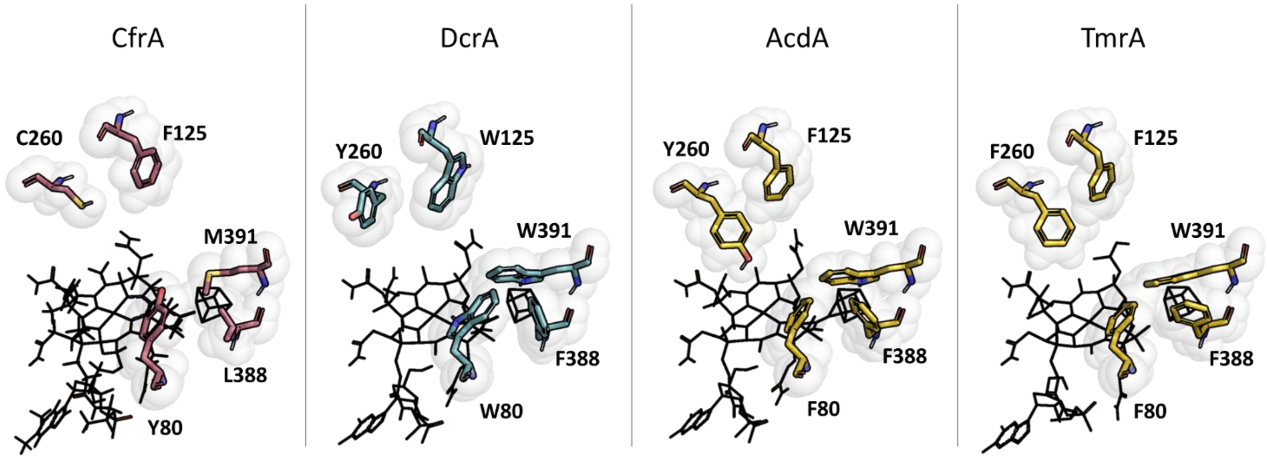

Figure S7. Active site configuration of CfrA (outer left), DcrA (inner left), AcdA (inner right) and TmrA (outer right).

Table S4. The steric strain of each of the five target active site residues in CfrA, DcrA, and the mutants. The steric strain (kcal/mol) is for the most favourable rotamer for the residue, the observed rotamer percent is in brackets. The colour of the values matches which residue the protein/mutant has indicated in the column header; black matches the CfrA residues, and blue values match the DcrA residues. The strain of Y256 is shown for two rotamers; toward and away from the active site, to demonstrate the various strain differences.

Enzyme	Y80 W80	F125 W125	C260 Y260	L388 F388	M391 W391	Y256 toward active site	Y256 away from active site
CfrA	19.34 (31.5%)	18.68 (65.7%)	20.51 (69.7%)	13.77 (34.1%)	18.50 (14.0%)	28.01 (76.8%)	39.74 (7.2%)
DcrA	14.74 (2.5%)	16.85 (48.5%)	21.02 (76.8%)	15.06 (16.5%)	22.22 (14.6%)	32.09 (22.3%)	25.57 (13.4%)
CfrA-5M	25.5 (26.5%)	31.96 (28.7%)	40.31 (39.5%)	26.61 (23.3%)	45.07 (13.5%)	80.86 (76.8%)	63.98 (13.3%)
CfrA-3M	24.66 (31.4%)	34.17 (65.7%)	32.03 (39.5%)	24.67 (23.3%)	40.21 (13.5%)	65.96 (76.8%)	56.87 (7.2%)
CfrA- Y80W-F125W	19.82 (26.5%)	30.17 (28.7%)	56.47 (69.7%)	16.60 (34.1%)	19.17 (14.0%)	41.83 (76.8%)	42.16 (13.3%)
CfrA-Y80W	19.26 (26.5%)	18.68 (65.7%)	20.51 (69.7%)	16.60 (34.1%)	18.80 (14.0%)	28.04 (76.8%)	41.36 (13.3%)
CfrA-F125W	19.34 (31.5%)	26.53 (28.7%)	46.95 (69.7%)	13.77 (34.1%)	18.50 (14.0%)	41.78 (76.8%)	42.11 (7.2%)
CfrA-C260Y	19.56 (31.5%)	28.28 (65.7%)	33.54 (39.4%)	13.77 (34.1%)	30.80 (14%)	56.49 (76.8%)	47.99 (13.3%)

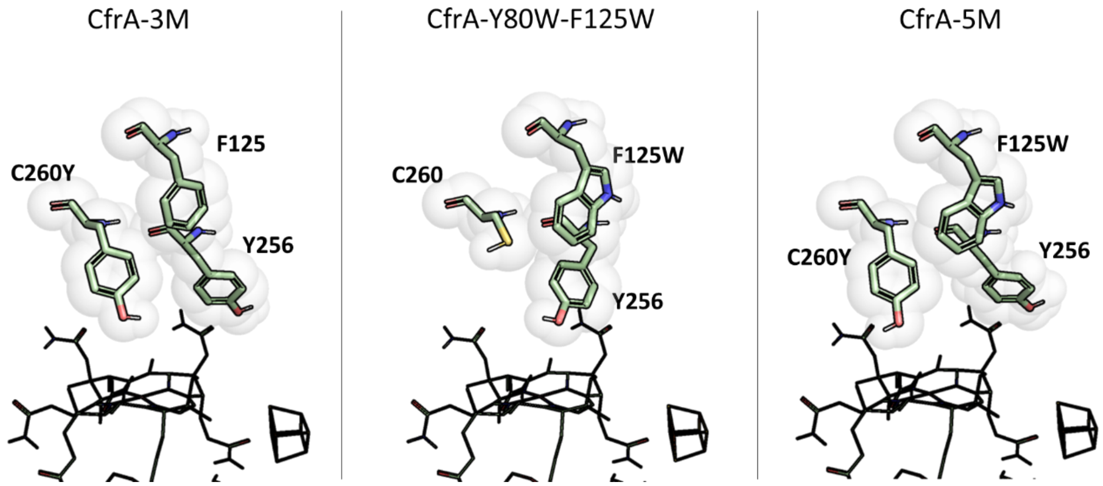

Figure S8. Predicted Y256 rotamer of CfrA-3M (left), CfrA-Y80W-F125W (center), and CfrA-5M (right).

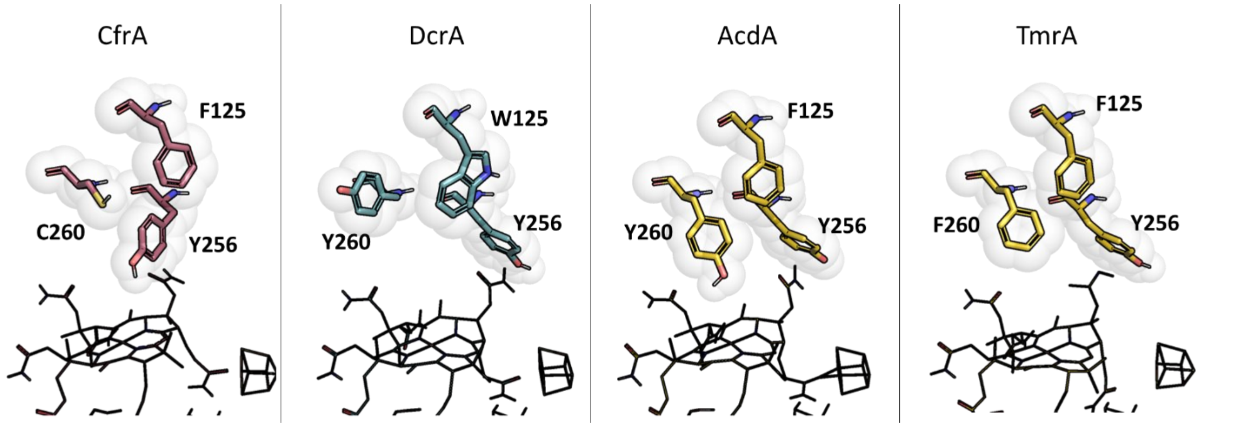

Figure S9. Predicted Y256 rotamer of CfrA (outer left), DcrA (inner left), AcdA (inner right) and TmrA (outer right).

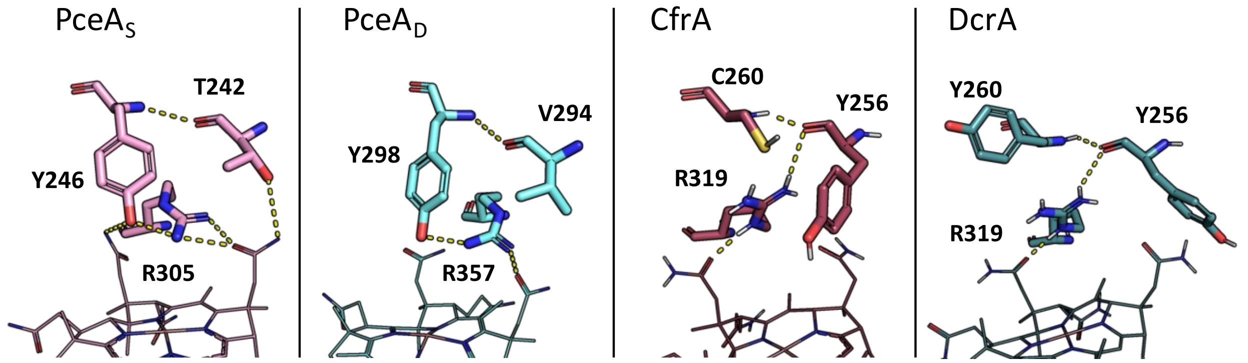

Figure S10. Predicted interactions of the conserved arginine with cobalamin (wire structure) and surrounding residues in crystal structures of PceA$_S$ from *Sulfurospirillum multivorans* (PDB ID: 4UR0) and PceA$_D$ from *Desulfitobacterium hafniense* TCE1 (8Q4H), and AlphaFold2 models of CfrA and DcrA (6, 7). Dashed lines indicate PyMOL predicted polar interactions within 3.5 Å.

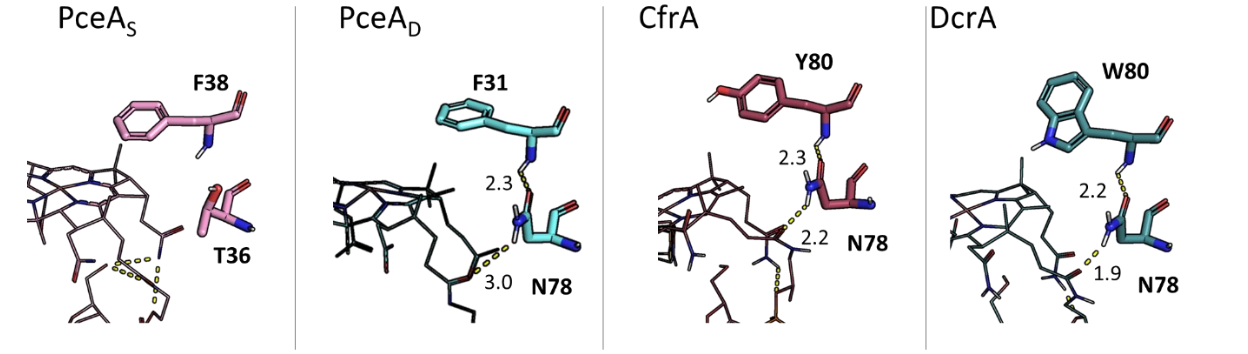

Figure S11. Predicted interaction cascade of residue 80 in crystal structures of PceA$_S$ from *Sulfurospirillum multivorans* (PDB ID: 4UR0) and PceA$_D$ from *Desulfitobacterium hafniense* TCE1 (8Q4H), and AlphaFold2 models of CfrA and DcrA (6, 7). Dashed lines indicate PyMOL predicted polar interactions within distance in angstroms.

Supplemental Text S5 – DcrA Mutants

In addition to DcrA-5M, which had the active site transplant from CfrA, two other DcrA mutants were produced that had various other changes in the active site and in the [4Fe-4S] vicinity. The CfrA mutant results revealed that the active site residues analyzed did have an effect on the substrate selectivity, but the activity was not as high as DcrA on 1,1-DCA and CfrA-5M still had considerable activity on CF and 1,1,1-TCA. Since 1,1-DCA has a greater energy barrier to reduction due to its lower reduction potential (E_0' = 397 mV) than CF (560 mV) and 1,1,1-TCA (561 mV) (8), we predicted that there might be differences in the structure that affect the electron transfer from the [4Fe-4S] clusters. It is known that the inner and outer coordination spheres of iron-sulfur clusters can change their redox potential and the idea of a redox potential optima has been suggested (9–12). These ideas may point towards DcrA having tuned its redox potential for the more difficult 1,1-DCA. Differences in the redox potential of each enzyme cannot be determined by this assay setup because they are all an electron donor (methyl viologen) with very low redox potential, and redox measurements are out of the scope of this study. Nevertheless, we predicted that variations around the [4Fe-4S] clusters between CfrA and DcrA may be contributing to the incomplete activity shift.

We produced the mutant DcrA-FeS, which has mutations R94V, T244V, A247D, and P316S. All except A247D are in the vicinity of either [4Fe-4S] cluster; A247D is located closer to the enzyme access channel. We predicted that if any of these residues had an effect on the redox potential and substrate selectivity, we would get a similar profile to CfrA-5M as DcrA-FeS retained all of the active site residues but has vicinal residues similar to CfrA. We also created DcrA-AS-FeS which had the mutations W80Y, W125F, and T244V; the first two are in the active site and the third is near the [4Fe-4S] clusters and DcrA is the only OG 97 with a proton-donor in that position. DcrA-AS-FeS we expected to behave more similarly to DcrA-3M as it would have the same active site.

The DcrA mutants did have some notable shifts in activity, but not large shifts in relative substrate preference (Figure S12 and Figure S13). DcrA-5M did artificially shift its substrate preference to be more similar to CfrA (Figure S12D), but the activity values are so low for this mutant that these mutations appear to be deleterious to activity rather. This is similar to what was seen in DcaA, with the opening of the active site diminishing all activity on smaller substrates (13), and the restriction of activity on the other substrates may be due to filtering earlier in the substrate channel which was not targeted with these mutations. However, we would not expect this to also limit the activity seen on 1,1,2-TCA as we do, which could suggest that the low solubility of the mutant caused the low observed activity.

Both DcrA-FeS and DcrA-AS-FeS had similar activity and a similar substrate profile to DcrA. While this was expected for DcrA-FeS, we do see a decrease in 1,1-DCA reduction and an increase in 1,1,2-TCA dihaloelimination similar to CfrA-5M. Further investigation is needed to determine the effect on the redox potential and whether this, in turn, affects the activity.

The lack of a change in DcrA-AS-FeS confounds the deterministic role of residues 80 and 125 on the activity as we saw in CfrA-Y80W-F125W. We predicted that DcrA-AS-FeS would have similar activity to CfrA-3M, but it is clear that this is not the case. This lack of change points to other influential residues earlier in the substrate access channel, which would lead to the early filtering of the larger substrates in DcrA-AS-FeS but leave the filtering at the mouth of the active site in CfrA-Y80W-F125W and no filtering for CfrrA-3M. The role of residues along the substrate access channel should also be investigated further.

Visualizations of the DcrA mutants are shown in Figure S15-Figure S17.

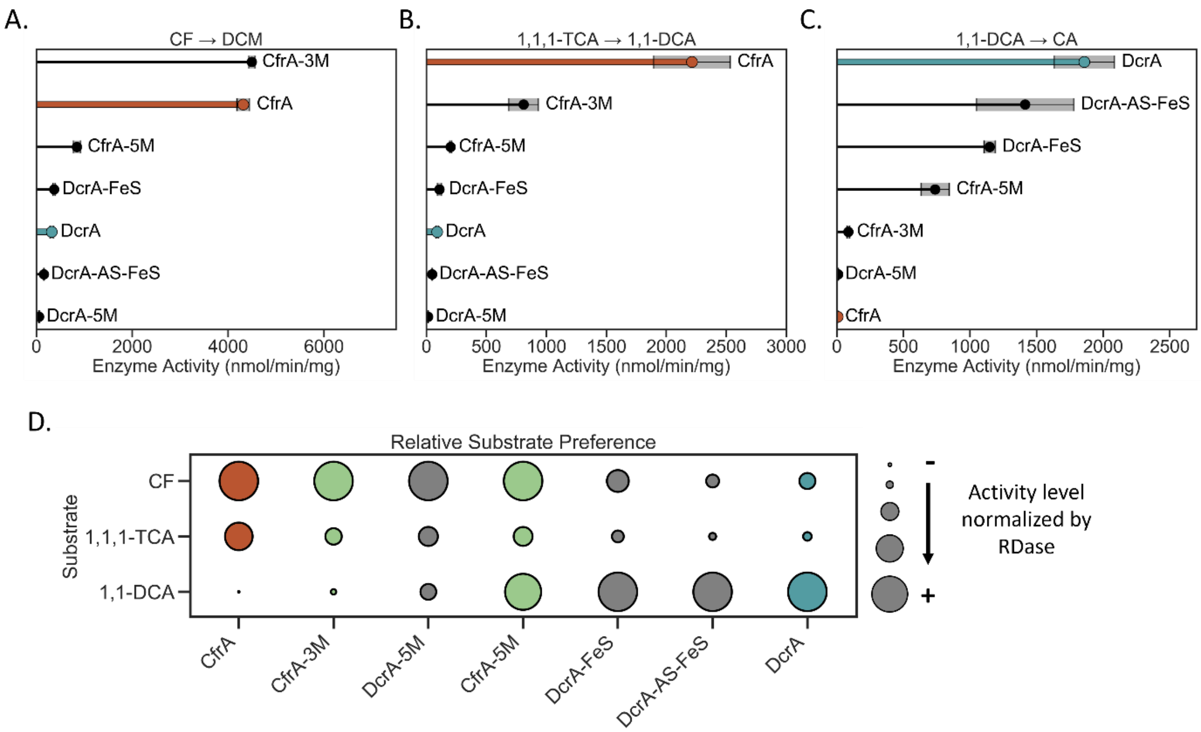

Figure S12. Dechlorination activity of DcrA mutants on (A) chloroform, (B) 1,1,1-trichloroethane, and (C) 1,1-dichloroethane after 1 hr. CfrA activity is highlighted in red, DcrA is highlighted in blue, and CfrA mutants in green (D) The activity of each enzyme scaled from 0 to the substrate with the highest activity to show the relative substrate preferences, larger circles indicate higher activity levels. Grey shading indicates the standard deviation between samples (n = 3, n = 4 for CfrA and DcrA). CF = chloroform, DCM = dichloromethane, TCA = trichloroethane, DCA = dichloroethane, CA = chloroethane.

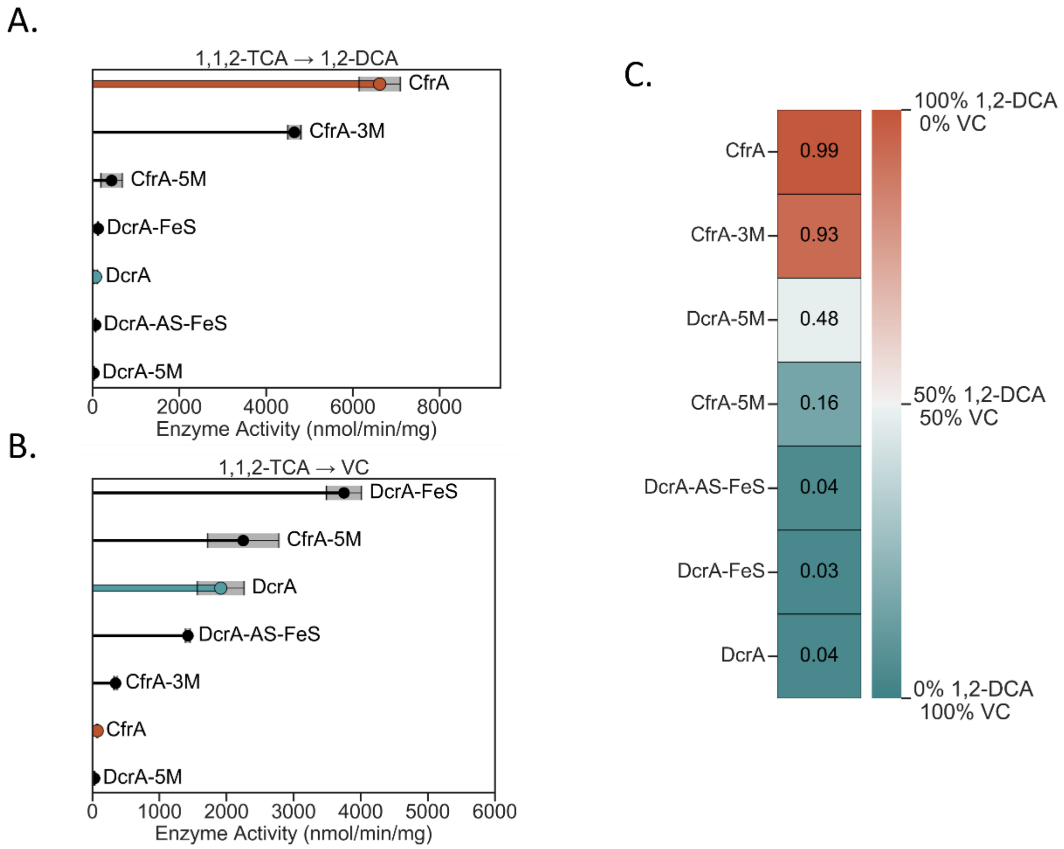

Figure S13. Dechlorination activity of DcrA mutants on 1,1,2-trichloroethane to produce (A) 1,2-dichloroethane or (B) vinyl chloride after 1 hr. (C) The proportion of the dechlorination product that went to 1,2-dichloroethane (more red) and vinyl chloride (more blue); the number in the square indicates the ratio of 1,2-dichloroethane product. CfrA activity is highlighted in red, DcrA in blue, and CfrA mutants in green. Grey shading indicates the standard deviation between samples (n = 3, n = 4 for CfrA and DcrA). TCA = trichloroethane, DCA = dichloroethane, VC = vinyl chloride.

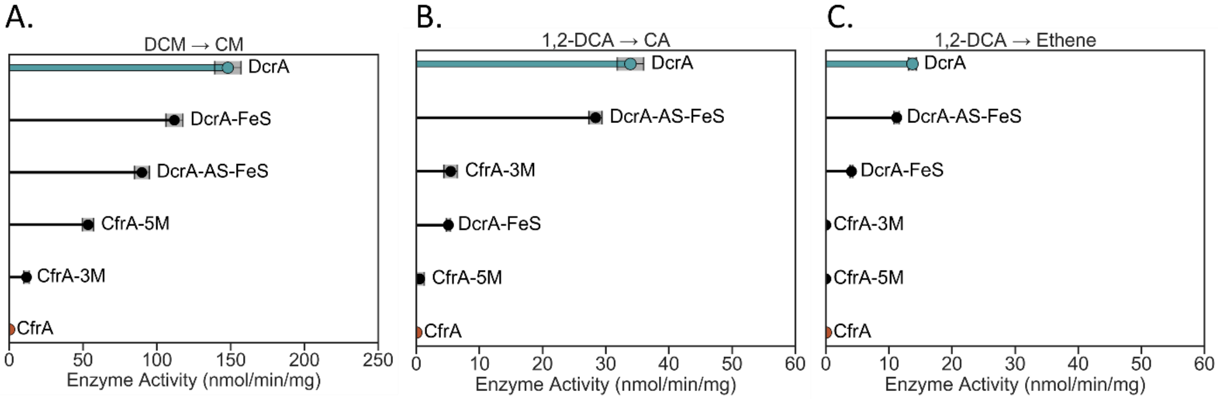

Figure S14. DcrA mutant dechlorination assay of dichloromethane (DCM; A) to chloromethane (CM) and 1,2-dichloroethane (DCA) to chloroethane (CA; B) and ethene (C) after 1 hr. CfrA is highlighted in red and DcrA in blue. Error bars are standard deviation between triplicate reactions.

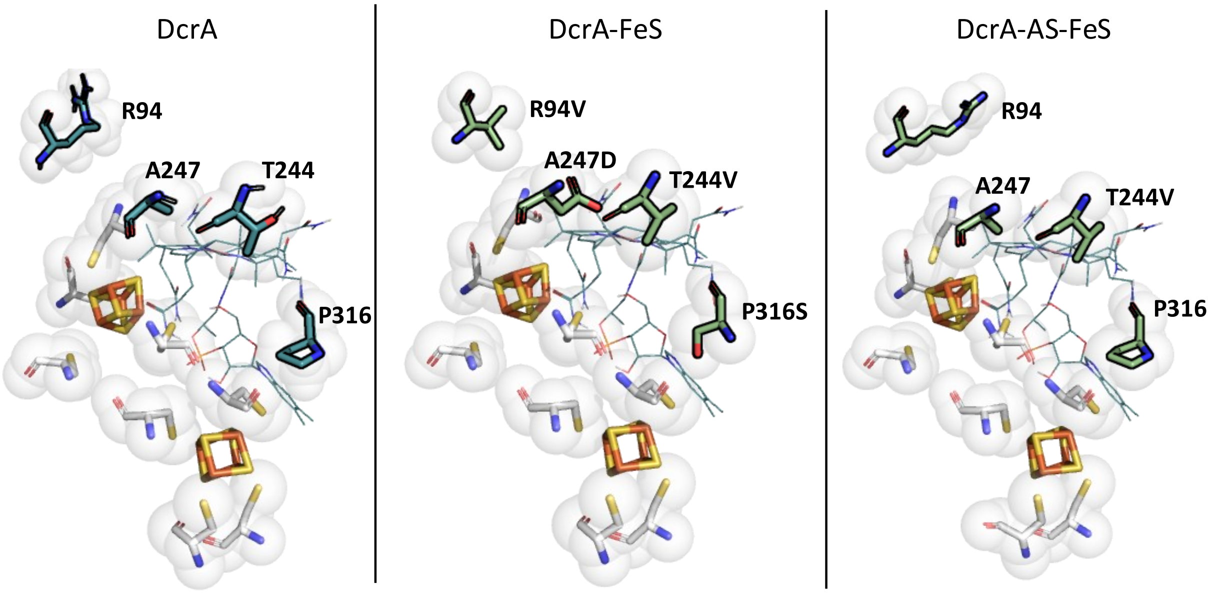

Figure S15. Residue differences around [4Fe-4S] for DcrA (left), DcrA-FeS (center), and DcrA-AS-FeS (right).

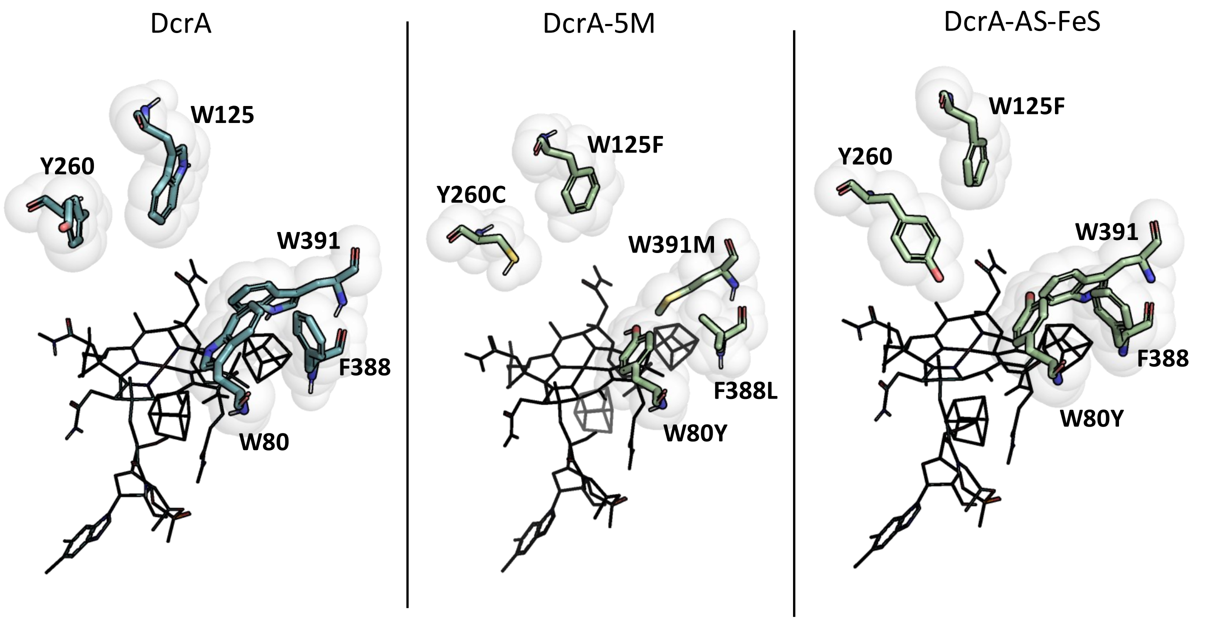

Figure S16. Active site configuration for DcrA (left), DcrA-5M (center), and DcrA-AS-FeS (right).

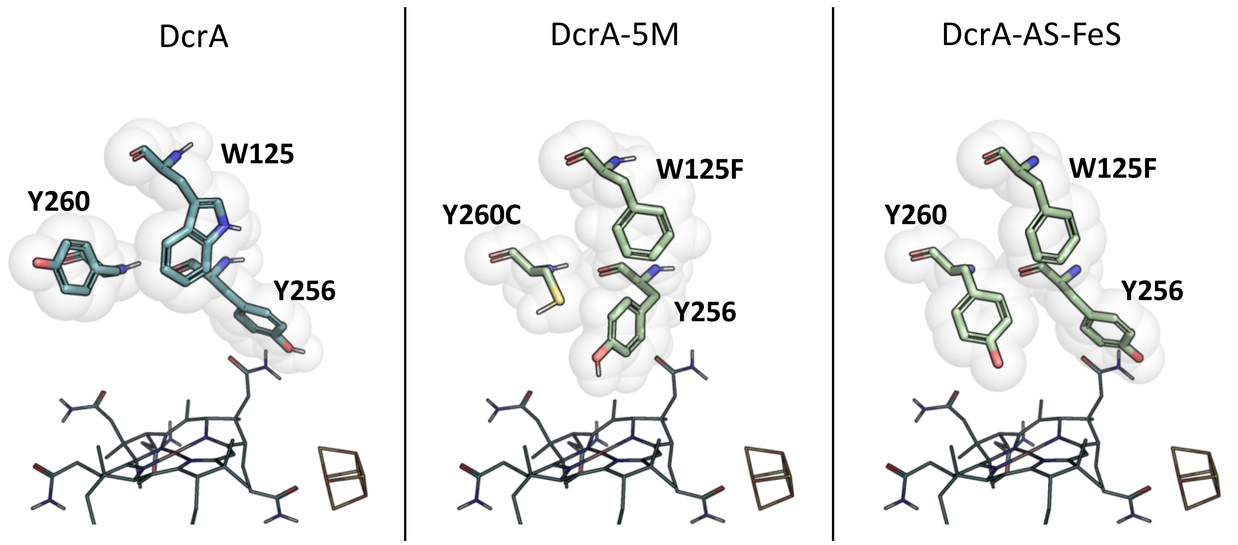

Figure S17. Predicted Y256 rotamer of DcrA (left), DcrA-5M (center), and DcrA-AS-FeS (right).

References

1. Bulka O, Picott K, Mahadevan R, Edwards EA. 2024. From *mec* cassette to *rdhA*: a key *Dehalobacter* genomic neighborhood in a chloroform and dichloromethane–transforming microbial consortium. Appl Environ Microbiol 90:e00732-24.

2. Soder-Walz JM, Wasmund K, Deobald D, Vicent T, Adrian L, Marco-Urrea E. 2023. Respiratory protein interactions in Dehalobacter sp. strain 8M revealed through genomic and native proteomic analyses. Environ Microbiol 25:2604–2620.

3. Picott KJ, Flick R, Edwards EA. 2022. Heterologous expression of active *Dehalobacter* respiratory reductive dehalogenases in *Escherichia coli*. Appl Environ Microbiol 88:e01993-21.

4. Lanz ND, Blaszczyk AJ, McCarthy EL, Wang B, Wang RX, Jones BS, Booker SJ. 2018. Enhanced solubilization of class B radical S-adenosylmethionine methylases by improved cobalamin uptake in *Escherichia coli*. Biochemistry 57:1475–1490.

5. Virtanen P, Gommers R, Oliphant TE, Haberland M, Reddy T, Cournapeau D, Burovski E, Peterson P, Weckesser W, Bright J, van der Walt SJ, Brett M, Wilson J, Millman KJ, Mayorov N, Nelson ARJ, Jones E, Kern R, Larson E, Carey CJ, Polat İ, Feng Y, Moore EW, VanderPlas J, Laxalde D, Perktold J, Cimrman R, Henriksen I, Quintero EA, Harris CR, Archibald AM, Ribeiro AH, Pedregosa F, van Mulbregt P, Vijaykumar A, Bardelli A Pietro, Rothberg A, Hilboll A, Kloeckner A, Scopatz A, Lee A, Rokem A, Woods CN, Fulton C, Masson C, Häggström C, Fitzgerald C, Nicholson DA, Hagen DR, Pasechnik D V., Olivetti E, Martin E, Wieser E, Silva F, Lenders F, Wilhelm F, Young G, Price GA, Ingold GL, Allen GE, Lee GR, Audren H, Probst I, Dietrich JP, Silterra J, Webber JT, Slavič J, Nothman J, Buchner J, Kulick J, Schönberger JL, de Miranda Cardoso JV, Reimer J, Harrington J, Rodríguez JLC, Nunez-Iglesias J, Kuczynski J, Tritz K, Thoma M, Newville M, Kümmerer M, Bolingbroke M, Tartre M, Pak M, Smith NJ, Nowaczyk N, Shebanov N, Pavlyk O, Brodtkorb PA, Lee P, McGibbon RT, Feldbauer R, Lewis S, Tygier S, Sievert S, Vigna S, Peterson S, More S, Pudlik T, Oshima T, Pingel TJ, Robitaille TP, Spura T, Jones TR, Cera T, Leslie T, Zito T, Krauss T, Upadhyay U, Halchenko YO, Vázquez-Baeza Y. 2020. SciPy 1.0: fundamental algorithms for scientific computing in Python. Nat Methods 17:261–272.

6. Jumper J, Evans R, Pritzel A, Green T, Figurnov M, Ronneberger O, Tunyasuvunakool K, Bates R, Žídek A, Potapenko A, Bridgland A, Meyer C, Kohl SAA, Ballard AJ, Cowie A, Romera-Paredes B, Nikolov S, Jain R, Adler J, Back T, Petersen S, Reiman D, Clancy E, Zielinski M, Steinegger M, Pacholska M, Berghammer T, Bodenstein S, Silver D, Vinyals O, Senior AW, Kavukcuoglu K, Kohli P, Hassabis D, Hassabis D. 2021. Highly accurate protein structure prediction with AlphaFold. Nature 596:583–589.

7. Hekkelman ML, de Vries I, Joosten RP, Perrakis A. 2023. AlphaFill: enriching AlphaFold models with ligands and cofactors. Nat Methods 20:205–213.

8. De Wildeman S, Verstraete W. 2003. The quest for microbial reductive dechlorination of C_2 to C_4 chloroalkanes is warranted. Appl Microbiol Biotechnol 61:94–102.

9. Stephens PJ, Jollie DR, Warshel A. 1996. Protein control of redox potentials of iron-sulfur proteins. Chem Rev 96:2491–2513.

10. Davidson VL. 2008. Protein control of true, gated, and coupled electron transfer reactions. Acc Chem Res 41:730–738.

11. Elliott SJ, Léger C, Pershad HR, Hirst J, Heffron K, Ginet N, Blasco F, Rothery RA, Weiner JH, Armstrong FA. 2002. Detection and interpretation of redox potential optima in the catalytic activity of enzymes. Biochim Biophys Acta 1555:54–59.

12. Zeamari K, Gerbaud G, Grosse S, Fourmond V, Chaspoul F, Biaso F, Arnoux P, Sabaty M, Pignol D, Guigliarelli B, Burlat B. 2019. Tuning the redox properties of a [4Fe-4S] center to modulate the activity of Mo-*bis*PGD periplasmic nitrate reductase. BBA - Bioenerg 1860:402–413.

13. Kunze C, Diekert G, Schubert T. 2017. Subtle changes in the active site architecture untangled overlapping substrate ranges and mechanistic differences of two reductive dehalogenases. FEBS J 284:3520–3535.
